# VOC injection into a house reveals large surface reservoir sizes in an indoor environment

**DOI:** 10.1073/pnas.2503399122

**Published:** 2025-09-22

**Authors:** Jie Yu, Pascale S. J. Lakey, Jenna C. Ditto, Han N. Huynh, Michael F. Link, Dustin Poppendieck, Stephen M. Zimmerman, Xing Wang, Delphine K. Farmer, Marina E. Vance, Jonathan P. D. Abbatt, Manabu Shiraiwa

**Affiliations:** ^a^Department of Chemistry, University of Toronto, Toronto, ON M5S 3H6, Canada; ^b^Department of Chemistry, University of California, Irvine, CA 92697-2025; ^c^Department of Energy, Environmental, and Chemical Engineering, Washington University in St. Louis, St. Louis, MO 63130; ^d^Cooperative Institute for Research in Environmental Sciences, University of Colorado, Boulder, CO 80309; ^e^National Oceanic and Atmospheric Administration, Chemical Sciences Laboratory, Boulder, CO 80309; ^f^National Institute of Standards and Technology, Gaithersburg, MD 20899; ^g^Department of Chemical Engineering and Applied Chemistry, University of Toronto, Toronto, ON M5S 3E5, Canada; ^h^Department of Chemistry, Colorado State University, Fort Collins, CO 80523; ^i^Department of Mechanical Engineering, University of Colorado, Boulder, CO 80309

**Keywords:** indoor air, VOC partitioning, surface reservoir, gas–surface interaction, contaminant exposure

## Abstract

The total partitioning capacity of indoor surface reservoirs determines the mechanism by which humans receive nondietary exposure to organic contaminants, via inhalation, dermal uptake, and dust ingestion. And yet, this capacity is largely unknown. Surface organic films are ubiquitously present but have very low partitioning volume being only 10’s of nanometer thick, whereas other surface reservoirs such as building materials and furnishings can be permeable or porous with large surface areas at the molecular level. Here, we assess the total partitioning capacity of volatile organic compounds (VOCs) in an indoor environment from the measured kinetics of VOC surface uptake after injection of compounds with variable volatility into a well-characterized, unoccupied test house. We show that the size of the indoor surface reservoirs is very large with an octanol-equivalent average thickness on the order of micrometers, indicating that permeable/porous materials such as painted surfaces and wood are likely the major surface reservoirs in the house rather than organic surface films. Large surface reservoirs result in compounds with octanol-air partition coefficients (*K*_OA_) larger than 10^5^ being predominantly partitioned to indoor surface reservoirs, making them hard to be removed via ventilation. This result significantly impacts our understanding of VOC fate and human exposure in indoor environments. With such a large partitioning capacity, organic contaminants will have much longer indoor residence times than previously predicted.

## Significance

Organic contaminants partition to indoor surfaces, giving rise to human exposure via dermal uptake and nondietary ingestion pathways. However, the total partitioning capacity of indoor surfaces has not been experimentally quantified in the built environment. By injecting various organic contaminants into the air of a test house, we determine the degree to which these contaminants partition to indoor surfaces. Our estimates of the total surface partitioning capacity are much larger than if the reservoirs are taken to be thin organic films on smooth, impermeable surfaces. This directly affects assessments of how humans are exposed to contaminants that undergo partitioning between indoor air and surfaces.

Surfaces available in the built environment strongly affect the mechanism by which humans are exposed to organic contaminants. In particular, we know that many volatile organic compounds (VOCs) undergo significant partitioning to large indoor surface reservoirs, whereas these molecules reside largely in the gas phase in the outdoor environment (1–5). This reduces the degree to which humans experience inhalation exposure but increases the rates of dermal uptake, which can occur upon touching a contaminated surface, and nondietary ingestion, especially for young children (6). Upon the introduction of organic chemicals into indoor spaces via cooking, cleaning, or the use of personal care and other consumer products, the connections between contaminant chemical properties and the degree of transport and multiphase partitioning behavior are still unclear. Controlled chamber studies have been conducted to demonstrate the uptake of gases to representative indoor surfaces such as carpet, glass, paint and wallboard (7–10). However, rapid real-time measurements of the transport and partitioning of a suite of chemicals in genuine residential indoor spaces are rare (11–13).

Indoor surfaces are highly complex, leading to challenges in representation of their partitioning capacity (14). An indoor surface incorporates not only the gas–surface interface but also pores and deposited particles. Additionally, thin organic films, 10’s of nanometers thick, are present on surfaces, arising from the deposition of semivolatile organic molecules (15–17). The underlying building or house furnishing materials are highly variable, with the rate of diffusion from the gas–surface interface extremely slow into impermeable materials, such as glass, but very fast (on the timescale of hours) into others, such as paint films (18).

The tendency to partition to indoor surfaces is frequently parameterized in terms of the octanol-air partition coefficient (*K*_OA_) which describes at equilibrium the degree to which a molecule partitions to octanol relative to air, where octanol is chosen as a reference compound because it participates in both polar and nonpolar intermolecular interactions with organic contaminants. The use of *K*_OA_ for describing multimedia partitioning processes in the environment has been long established (19–22) and a recent study has shown that the effective whole-house partitioning coefficient is linearly proportional to *K*_OA_ (23). In particular, Weschler and Nazaroff used this framework to describe how the equilibrium partitioning of semivolatile organic compounds (SVOCs) proceeds indoors when only surface films 10’s of nm thick are present (see figure 4 of their paper), predicting that molecules with log *K*_OA_ values larger than about 7 will largely exist on the surface (4, 15–17). Partitioning to deeper or porous reservoirs would adjust this value. As stated, the justification of using *K*_OA_ as the partitioning parameter comes from its extensive use in modeling outdoor contaminant behavior. However, specific partitioning studies using indoor air have recently demonstrated that the degree of cloth-air partitioning scales with the log *K*_OA_ of the chemical of interest (24, 25). As well, the early work of Singer et al. showed that the kinetics of uptake of VOCs in furnished rooms scale with their *K*_OA_ values (5). Despite these studies, a major constraint in our ability to model VOC air-surface partitioning remains that we do not know the total size of the surface reservoirs in indoor environments, i.e., their partitioning capacity. Surface reservoirs may include organic films and part or all of the underlying material. Within the *K*_OA_ framework, this corresponds to not knowing the volume of octanol that describes the sorption properties of the indoor surface reservoirs.

The persistence and spatial/temporal variability of chemicals in indoor spaces of VOCs are now being addressed, aided by recent studies that have used fast-time-response (e.g., 1 min) measurements (3, 26–29). For example, modeling of the spatial and temporal scales of short- and long-lived gas-phase species and particles of various sizes revealed dependence on reactivity and ventilation conditions (26). Nevertheless, for VOCs such analyses are limited by lack of knowledge of the indoor surface partitioning capacity. For example, the rate at which the concentration of a gas emitted within a building will become uniform throughout an indoor space is dependent on not only the air mixing timescale but also on the degree to which the indoor surface reservoirs act as partitioning sinks for the gas.

**Fig. 1.**
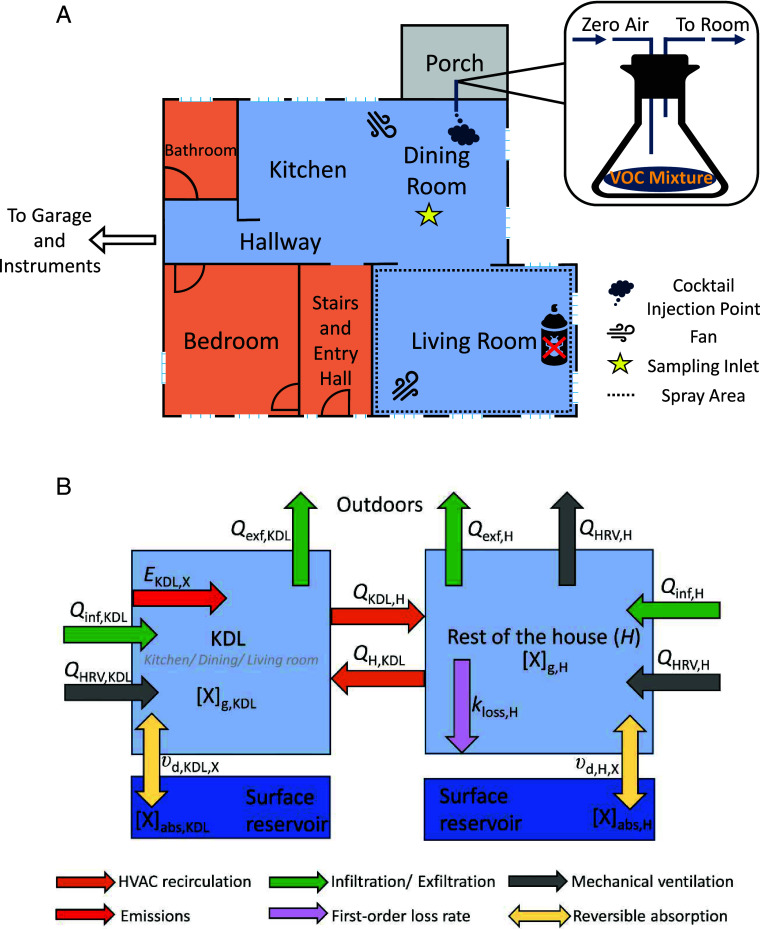
(*A*) Floor plan for the first floor of the house. Locations of the *CASA* cocktail and the insecticide emission sources and the mixing fans are noted. An illustration of the *CASA* cocktail injection set up in the isolated porch area is also shown. (*B*) Schematic diagram of the two-box model. Dark blue boxes represent the surface reservoirs. The open kitchen-dining-living room area is denoted as “KDL.”

The goal of this work is to make a quantitative assessment of the VOC partitioning capacity of indoor surfaces in the built environment. As part of a comprehensive indoor air study in a residential test house—The Chemical Assessment of Surfaces and Air (CASA) (11)—real-time gas-phase measurements were conducted for two gas emission events (*SI Appendix*, Text S1) meant to mimic the daily use of consumer products that contain VOCs. One event involved the simultaneous addition of relatively volatile species (referred to as the *CASA* Cocktail) and the other involved an insecticide spray which contains some lower volatility and higher *K*_OA_ molecules than the *CASA* Cocktail. The locations within the test house of each emission source are indicated in Fig. 1*A*. The temporal profile of the VOCs was measured using a proton-transfer-reaction mass spectrometer. Taking advantage of the characterized air handling flows within the test house, we simulated the decay profiles of specific VOCs using a two-box model that is shown schematically in Fig. 1*B*, thereby investigating the dependence of surface partitioning behavior on their *K*_OA_ values (*SI Appendix*, Text S2). This leads to an evaluation of the total partitioning capacity of the house. An estimation of persistence in surface reservoirs was applied to a broad range of indoor pollutants to assess their dependence on surface reservoir size, log *K*_OA_, and house ventilation rates. Note that in this work, we refer to all gases studied as VOCs, based on the criterion of Weschler and Nazaroff (4), even if these species demonstrate some degree of semivolatile partitioning behavior with large surface reservoirs.

## Results and Discussion

### Measured and Model Decay Profiles.

**Fig. 2.**
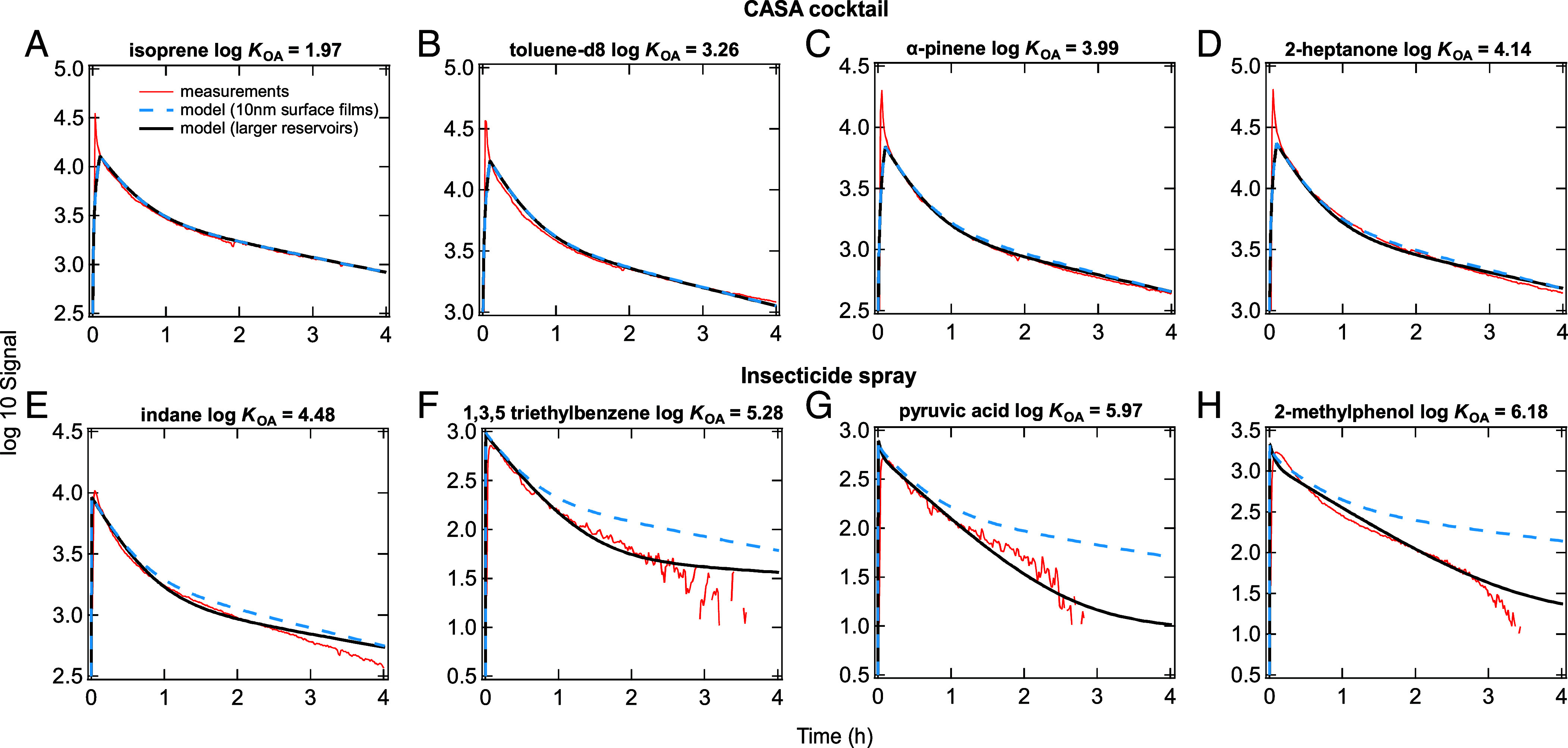
Gas-phase measurements in the KDL (red lines) of selected compounds released into the KDL during the *CASA* (*A*–*D*) cocktail experiment and (*E*–*H*) pesticide experiment. Outputs from the kinetic model are shown assuming 10 nm surface films throughout the house (dashed blue lines) and larger surface reservoirs with an octanol-equivalent average thickness of 200 nm in the KDL and 8 μm in the rest of the house (black lines). Rms errors (RMSE), which indicate average difference between modeling and measurements, are shown for the model simulations with the larger reservoirs and calculated between 30 min and 3 h to exclude data where the house is not well-mixed and the signal is low with greater uncertainty.

The gas-phase measurements and modeling of four selected *CASA* cocktail compounds (isoprene, toluene, α-pinene, 2-heptanone) in the open concept kitchen-dining-living room area (KDL) are presented in Fig. 2 *A*–*D*. Data for all cocktail compounds are provided in *SI Appendix*, Fig. S1. As expected, the mixing ratios (i.e., mole of analyte/mole of air molecules) of all species declined after injection due to mixing from the KDL to the rest of the house, partitioning to indoor surfaces, and ventilation. Note that the measured decay rates are not kinetically first order, due to incomplete mixing within the KDL over the first ~30 min and the time dependence of transport both into and from the rest of the house. The injection of a tracer, CO_2_, also shows this temporal behavior (see *Discussion* below and *SI Appendix*, Fig. S3).

We conducted kinetic box modeling by considering interzonal flow in the house, air change with the outdoors, emissions, and surface partitioning. The deposition velocity is treated by considering gas-phase diffusion across a surface boundary layer, surface accommodation, and bulk diffusion to organic films or surface materials using an effective mass accommodation coefficient (30). This *α*_eff_ method is compared with kinetic multilayer modeling which explicitly treats all detailed mass transport processes including bulk diffusion in surface reservoirs (31), providing consistent results and hence validating the α_eff_ method (*SI Appendix*, Fig. S4 see *Materials and Methods* and *SI Appendix* for details). Two scenarios were simulated: The first was an indoor-representative 10 nm octanol-equivalent average thickness of surface films accessible for partitioning everywhere in the house (15–17) and the second implemented a much larger surface reservoir, which describes the data most closely, with an average octanol-equivalent thickness of 200 nm in the KDL and 8 μm in the rest of the house. For these base case simulations, surface uptake of VOCs was assumed to be governed by gas-phase diffusion with the effective mass accommodation coefficient set to unity by assuming no bulk diffusion limitations (18). Potential reasons for the differences in the reservoir thicknesses between the two areas are discussed later. Note that these octanol-equivalent average thicknesses are conceptual, being derived from the octanol volume used in the simulation and the geometric surface area-to-volume ratio for the house (*SI Appendix*, Table S2). True organic surface film thicknesses in the house will depend on partitioning properties and the morphology of indoor surfaces at a microscopic scale. In addition, the size of the available reservoir may also be affected by the timescale of experiments as diffusion may occur deeper into the materials over longer time periods.

All *CASA* cocktail compounds are relatively volatile with log *K*_OA_ values in the range of 2.0 to 4.3. We can reproduce measured time series in the KDL using measured values of interzone and indoor-to-outdoor air flow rates in the two-box kinetic model, showing that all measured cocktail compounds had similar decay rates. This is because these volatile compounds do not partition significantly into indoor surfaces (*SI Appendix*, Fig. S5 *A* and *B*). For example, simulations predict that only up to 0.5% of 2-heptanone molecules and 0.004% of isoprene molecules in the KDL partition into surface reservoirs.

The two-box model underestimated the initial peak mixing ratios for all compounds likely because of the model assumption of homogeneous mixing within the KDL and potentially nonconstant emissions of the injection. Postcampaign CO_2_ injections were performed to obtain more accurate air flows between zones from each injection location performed during the *CASA* study (*SI Appendix*, Text S4 and Fig. S3). Fitting to the CO_2_ measurements over the 1 to 4 h timescale allowed the interzone airflows (*Q*_H,KDL_ and *Q*_KDL,H_) to be determined. We assume that the measurements of CO_2_ and VOCs at each location are representative of those mixing ratios, as it has been demonstrated that long-lived species including CO_2_ and VOCs are mostly well-mixed in a room and zonal scale and is investigated in more detail in the SI (26). One observation is that all VOCs in the cocktail experiments decayed at approximately the same rate as each other and at long timescales, but decayed at slightly faster rates than CO_2_ (*SI Appendix*, Fig. S3). The reason for this difference in decay rates remains unclear but might be explained by a transport-limited partitioning process to a specific location in the house or to a deep reservoir or sink, with only CO_2_ being transported back into the KDL. It could also be caused by different sampling locations of CO_2_ and the VOCs if the indoor-to-outdoor mass transport is highly spatially specific. This could indicate limitations in the assumption to represent the entire volume of the KDL with a single measurement. To account for this loss, a first-order VOC loss rate (*k*_loss_) of 0.12 h^−1^ was included in the model for all VOCs (*SI Appendix*, Fig. S1).

Similarly, Fig. 2 *E*–*H* shows the behavior of sample compounds that are part of the insecticide spray; decay plots for all insecticide compounds are provided in *SI Appendix*, Fig. S2. A 30-min O_3_ injection was conducted during the observation period, resulting in the O_3_ reaching a maximum 50 ppb (32). Minor impacts on the gas-phase decay profiles are expected, only for the most reactive compounds (*SI Appendix*, Text S5). The log *K*_OA_ values of the compounds in the insecticide spray range between 4.0 and 6.2. We observed that compounds with smaller log *K*_OA_ values (insecticide, log *K*_OA_ = 4.0 to 4.5) decay at approximately the same rate which is similar to the CASA Cocktail compounds (log *K*_OA_ = 2.0 to 4.3). However, those with higher log *K*_OA_ values (>5) decayed at a faster rate, indicating stronger partitioning to the surface reservoirs.

With the assumption of 10 nm octanol-equivalent surface films on all surfaces available for partitioning, an underestimation of the measured decay was observed for compounds with higher log *K*_OA_ values (dashed blue lines in Fig. 2). Alternatively, by increasing the average reservoir thickness to 200 nm in the KDL and 8 μm in the rest of the house (with 10% variation, see *SI Appendix*, Table S2), the model aligned better with the measurements (solid black lines in Fig. 2) when assuming no bulk diffusion limitations. This supports the assumption that species with higher log *K*_OA_ values partition more readily into the reservoirs (*SI Appendix*, Fig. S5 *C* and *D*).

While this work does not fully elucidate the mechanism of sorption, these increased surface reservoir volumes indicate that permeable materials in the house, such as painted surfaces and wood, as well as concrete in the basement, are possibly the major surface reservoirs in the house rather than organic films on smooth impermeable surfaces, which tend to have average thicknesses on the order of only tens of nanometers. It is also possible that sorption is to highly porous indoor materials, that may or may not be coated with thin organic films, greatly increasing their effective partitioning capacity. In the study presented by Singer et al. (5), measurements of VOCs injected into a furnished room also led to observations whereby the sorption rate constant correlated with the log *K*_OA_ values of the gases. We note that the *k*_loss_ decay is negligible compared to the rates of partitioning into surface reservoirs for compounds with higher log *K*_OA_ values (above 5, *SI Appendix*, Fig. S2).

The difference in the model input for the average octanol-equivalent thicknesses of the surface reservoirs in the KDL and the rest of the house was necessary to reproduce the measurements for the higher log *K*_OA_ compounds in the insecticide spray. A sensitivity test performed for pyruvic acid demonstrated such reasoning. A large surface reservoir in the KDL caused rapid partitioning into the reservoir followed by a slow release back into the gas phase (*SI Appendix*, Fig. S6). A large reservoir is required in the rest of the house to decrease gas-phase mixing ratios and limit compounds being transported back to the KDL (*SI Appendix*, Fig. S6). The sensitivity of the mixing ratios of two different VOCs to variations in the thicknesses of the surface reservoirs in the KDL and rest of the house are shown in *SI Appendix*, Fig. S7. There are a number of reasons why the modeled surface reservoir sizes may be different between these spaces, perhaps due in part to the types of surfaces in the rooms and the prior activities within them. For example, there is a lot of exposed wood and concrete in the basement. Another possibility is that injected compounds may not fully interact with the KDL walls before they start mixing to the rest of the house. A computational fluid dynamics model would be required to better investigate whether flow patterns can account for the difference in the apparent surface reservoir sizes. Also, sensitivity tests indicated that increasing the surface-to-volume ratio ($\frac{S}{V}$) and similarly decreasing the average octanol-equivalent surface reservoir thickness by the same factor also resulted in a model that fit to the measurements, shown in Fig. 2, reasonably.

The experimental data are reproduced well (Fig. 2) with no bulk diffusion limitations in the surface materials (*α*_eff_ = 1). The simulation results from the kinetic multilayer model show almost identical results in the fitting to the data with *D*_b_ ≥ 5 × 10^−11^ cm^2^ s^−1^ (*SI Appendix*, Fig. S8). These results suggest that bulk diffusion limitations are not significant over the timescales of the experiments compared to other processes such as gas-phase mass transport between the different parts of the building and out of the building. We cannot exclude the possibility of larger bulk diffusion limitations effectively reducing the size of the surface reservoirs in the KDL leading to an underestimation in the KDL in our modeled reservoir thickness. This is shown in *SI Appendix*, Figs. S9 and S10 where the data have been fitted reasonably well with an average octanol-equivalent thickness of 8 μm throughout the house including the KDL but with a bulk diffusion coefficient of 10^−13^ cm^2^ s^−1^ in the KDL and >10^−11^ cm^2^ s^−1^ elsewhere. This bulk diffusivity is consistent with the viscosity of an organic film observed in the HOMEChem study (33). This may indicate different viscosities of materials in the different regions of the house or slightly more porous materials in the rest of the house compared to the KDL, resulting in the apparent different reservoir thicknesses if bulk diffusion limitations are not considered. It should also be noted that the octanol-equivalent reservoir thickness in the rest of the house cannot be smaller than 8 μm as a large partitioning volume is required to replicate the decays of higher log *K*_OA_ compounds. Additionally, although there are no modeled reactions in the surface reservoirs, sensitivity tests indicated that the measurements could be replicated with thinner surface reservoirs if these occurred. Finally, as there is a codependency between the surface reservoir thickness and bulk diffusivity, we are unable to accurately determine either value with the available dataset. Future measurements of thickness and viscosity/bulk diffusivity of surface films will be critical to further constrain the model.

The kinetic model was used to investigate the kinetics of surface uptake for the compounds with different log *K*_OA_ values and to determine the ratio of the molecules in the gas phase compared to those in the surface reservoirs (*SI Appendix*, Fig. S5). Overall, equilibrium between molecules in the gas phase and in surface reservoirs takes longer to achieve for compounds with higher log *K*_OA_ values and for thicker films (*SI Appendix*, Fig. S5). However, it should be noted that the model estimates of equilibration time are lower limits, as the internal mixing within the different zones of the house takes 5 to 15 min and could delay equilibrium being established. Once equilibrium has been reached, the ratio of the molecules in surface reservoirs compared to the gas phase can be estimated using the equilibrium ratio: $r_{e}=\frac{SLK_{OA,X}}{V}$ (*SI Appendix*, Fig. S11 and Tables S2 and S3) where *S* is the surface area, *L* is the reservoir thickness and *V* is the volume of the indoor space.

Although this paper focuses primarily on the size of the surface reservoirs, we note that this factor can affect the degree of temporal and spatial gradients in an indoor space after a short-lived VOC injection, with these gradients being steeper when the reservoirs are large. Note that after one hour, we do not anticipate significant spatial gas-phase concentration gradients, as even the compounds with higher partitioning coefficients are expected to be well-mixed over a distance of tens of meters (*SI Appendix*, Fig. S12). See *SI Appendix*, Text S3 and Figs. S12 and S13 where this relationship is explored.

### Persistence of Indoor Pollutants in Surface Reservoirs.

**Fig. 3.**
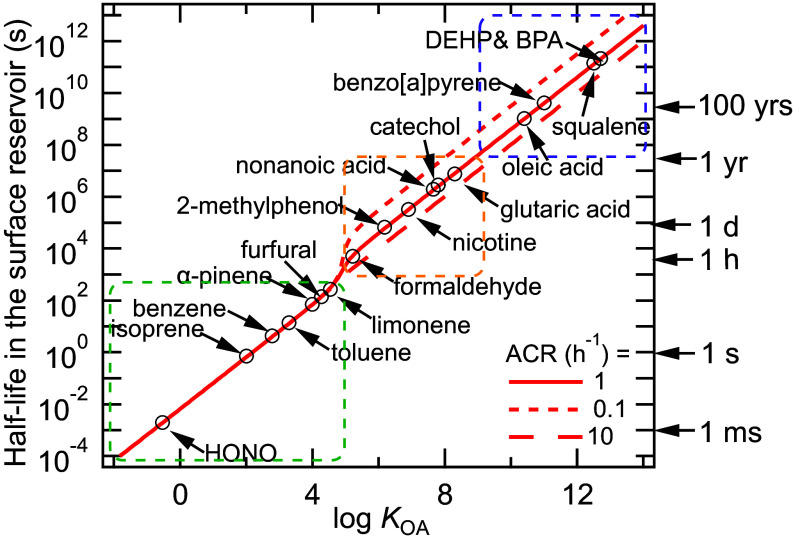
Half-life in the surface reservoir ($t_{\frac{1}{2},surf}$) of various compounds (with an average octanol-equivalent thickness of 8 μm) in the rest of the house as a function of the log *K*_OA_ value of the compound in the absence of reactions. *υ*_d_ is set to 3 m h^−1^ for all simulations and three different air change rates (ACR) of 0.1 (short dashed line), 1 (solid line), and 10 h^−1^ (long dashed line) were used in the kinetic model. The log *K*_OA_ values for specific compounds are taken from the literature (3, 29, 35, 36). See text for description of the colored dotted line boxes. For compounds with log *K*_OA_ ≤ 4.5 the half-life is controlled by mass-transport across the boundary layer. For compounds with log *K*_OA_ values ≥ 5 equilibrium is reached between the gas phase and the surface reservoir if the ACR is low leading to the half-life being controlled by the rate of indoor-to-outdoor transport.

Motivated by recent studies on the persistence of smoke-related VOCs indoors (29) and the residual impacts of wildfire on urban residential homes (34), the model was used to characterize the timescales that indoor pollutants will be removed from surface reservoirs in this test house under uniform concentration conditions, after a period of gas–surface partitioning. With the best-fit average octanol-equivalent surface reservoir thickness obtained previously in the rest of the house (8 μm) and with no bulk diffusion limitations (*α*_eff_ = 1), Fig. 3 illustrates the time for the concentration of species in a surface reservoir to decrease by half ($t_{\frac{1}{2},surf}$) when the indoor gas-phase concentration is set to zero. Results are plotted for a broad spectrum of common indoor acids, VOCs, and SVOCs as a function of their log *K*_OA_ values. Three different air change rates (ACR) of 0.1 (short dashed line), 1 (solid line), and 10 h^−1^ (long dashed line) were considered. This calculation does not consider the decay contributed from potential reactions in the surface reservoirs, thus the estimates are the upper limit of $t_{\frac{1}{2},surf}$ for each compound. A comparison of $t_{\frac{1}{2},surf}$ and the expected loss rate by chemical reactions can be used to determine whether chemical reactions or partitioning to the gas phase will be the main removal pathway (35), but that is beyond the scope of our current work.

For compounds with log *K*_OA_ ≤ ~4.5 (in the green dashed box), such as HONO, $t_{\frac{1}{2},surf}$ is less than one hour indicating that these compounds would be predominantly in the gas phase. For compounds with log *K*_OA_ ≤ ~4.5, $t_{\frac{1}{2},surf}$ is independent of ACR, as these compounds partition rapidly to the gas phase to reach 50% of their concentration in the reservoir, but do not reach equilibrium with the gas phase on the timescales of the half-lives (37). $t_{\frac{1}{2},surf}$ of HONO is very short on the timescale of 1 ms. Field observations have revealed a substantial abundance of HONO in indoor reservoirs as very rapid air-surface partitioning of HONO was observed upon enhanced ventilation such as window opening (3) Its very low *K*_OA_ and short lifetime in organic surface reservoirs (1 ms) imply that HONO mostly resides in polar (e.g., water) surface reservoirs, likely in the form of nitrite (36). Residing in the organic surface reservoirs, very short $t_{\frac{1}{2},surf}$ of some VOCs (green dashed box) including isoprene, α-pinene, limonene, benzene, and toluene indicate that these volatile chemical products that are applied as personal care products and cleaning agents in indoor environments would undergo indoor-to-outdoor transport easily, contributing to photochemical ozone formation and serving as precursors for secondary organic aerosols in the outdoor atmosphere (38).

As the log *K*_OA_ value increases from 5 to 9 (orange dashed box), $t_{\frac{1}{2},surf}$ increases significantly from less than 1 h to up to one year. Common indoor pollutants in this range of log *K*_OA_ include species often contained in insecticides (e.g., 2-methylphenol), cigarette smoke (e.g., nicotine), and wildfire smoke (e.g., glutaric acid and catechol). The lifetime of these compounds indoors can be extended via partitioning to the surface reservoir as modulated by ACR. Higher ACR, which may be achieved by opening windows or through mechanical ventilation, leads to shorter $t_{\frac{1}{2},surf}$, because once indoor compounds partition from the surface reservoir to the gas phase as controlled by gas diffusion across the boundary layer, they would be removed from indoor air more quickly before repartitioning to the surface reservoir. At low ACR, $t_{\frac{1}{2},surf}$ is controlled by the rate of indoor-to-outdoor transport as equilibrium is reached between the gas phase and the surface reservoir. Due to their long lifetime in the reservoir, these compounds will be removed via air change to the outdoors after partitioning to the gas phase. This explains why the smell of tobacco smoke (including nicotine) persists indoors even after a smoker leaves the room, as the residue of tobacco smoke that sticks to the surface reservoir, known as the “thirdhand smoke” (39), will partition back to the gas phase slowly. Chemical transformation of nicotine can generate products that can be carcinogenic and exposure to such compounds persist due to their overall long lifetime indoors (40, 41). Additionally, long $t_{\frac{1}{2},surf}$ are consistent with why some compounds persist once houses and buildings are exposed to wildfire smoke. While smoke contains a variety of compounds, some of them (e.g., furfural) may be easily removed by ventilation. However, for many smoke-related compounds (e.g., log *K*_OA_ > 5), regular and enhanced ventilation would be insufficient for the removal from surfaces. Surface cleaning activities such as vacuuming, mopping, and dusting are necessary to physically remove them from surface reservoirs (29).

Finally, compounds with very high log *K*_OA_ > 9 (purple dashed box) are expected to have $t_{\frac{1}{2},surf}$ longer than years, indicating that it is essentially impossible to remove them from buildings via ventilation. Field observations have revealed that squalene and its ozone reactivity persist in unoccupied homes (28), indicating that squalene is partitioned into surface reservoirs, perhaps buried in skin flakes; its overall lifetime is likely dominated by multiphase reactions. For oleic acid and benzo[a]pyrene which originate in part from cooking, their fate indoors is also largely determined by chemistry, in which multiphase reactions with indoor oxidants such as ozone have been found to contribute to their indoor loss (42, 43). Bisphenol A (BPA) and phthalates such as diethylhexyl phthalate (DEHP) are used as plasticizers, which may persist in surface reservoirs such as house dust, are endocrine disruptive chemicals and can exacerbate asthma and allergic symptoms (44, 45).

The size of the average octanol-equivalent surface reservoir will vary widely in different homes and buildings, where surface materials are different, and a small surface reservoir would lead to much shorter timescales. *SI Appendix*, Fig. S14 includes the estimation of $t_{\frac{1}{2},surf}$ if assuming a thinner 10 nm thick surface reservoir in the rest of the house, a typical thickness of indoor surface films. $t_{\frac{1}{2},surf}$ of a compound will be significantly reduced if it only resides in a relatively small surface reservoir. Large turbulence flows may slightly reduce $t_{\frac{1}{2},surf}$ (see *SI Appendix*, Fig. S15 for sensitivity simulations with various convective mass transfer coefficients). $t_{\frac{1}{2},surf}$ can be prolonged to hours and days in the presence of significant bulk diffusion limitations (*D*_b_ <10^−11^ cm^2^ s^−1^), as shown in sensitivity simulations in *SI Appendix*, Fig. S16. In conclusion, the interplay between partitioning, air-exchange, bulk diffusion and chemical reactions in the surface reservoirs will influence the persistence of compounds indoors and their concentrations in the gas phase and reservoirs.

### Implications for the Indoor Environment.

While it has been known for a long time that indoor surfaces are important reservoirs for many organic contaminants (3, 4, 24, 25, 46, 47), the degree to which these molecules are partitioned between the gas phase and the complex array of indoor surface materials has yet to be established. In particular, lab-based chamber studies have been conducted with a single type of surface material but there have been few measurements that assess the kinetics and degree of gas-to-surface partitioning in genuine indoor environments (3, 5, 29). With the development of online monitoring instrumentation, real-time measurement of airborne compounds has become available. A previous measurement in a test house has shown rapid replenishment of gas-phase VOCs from indoor surfaces after enhanced ventilation periods, demonstrating that dynamic gas–surface partitioning controls the gas-phase mixing ratios of many VOCs (3).

Building upon these past studies, this work uses measurements after VOC injections into a test house and a constrained two-box kinetic model to demonstrate the dependence of the gas-phase temporal decay on the chemical partitioning properties of the injected molecules. While another study in the same test house illustrated the long-term persistence of lower-volatility compounds in a smoke impacted setting (29), the present work demonstrates that the kinetics of the initial uptake process are determined by the log *K*_OA_ values of the injected molecules (5). Most significantly, the model fits of the experimental data yield an assessment of the partitioning capacity of indoor surface reservoirs.

The total partitioning capacity described by the model is in terms of the volume of a weakly polar organic compound, i.e., octanol. From this volume and the geometric surface area-to-volume ratio of the house, we find that an octanol-equivalent film thickness of 8 μm through much of the house is required to describe the data. To put this value into perspective, a typical thickness of organic films that develop on indoors surfaces is 10’s of nanometers, i.e., between 2 and 3 orders of magnitude thinner. This implies that these thin organic films on impermeable surfaces, while important for reactivity, are unlikely to dominate the overall partitioning capacity of an indoor space. Rather, the highly porous and permeable surfaces that are present in the building materials and furnishings will dominate partitioning, via both absorptive and adsorptive processes. Given the extremely large variability in the chemical nature, permeability, and specific surface area of different indoor surfaces (14), this total partitioning capacity has not been previously quantified in a genuine indoor environment. It should also be noted that the NIST house had a typical surface-to-volume ratio (2.0 m^−1^) for an unoccupied, unfurnished house (48). Furnishings can increase the surface-to-volume ratio by up to about a factor of two, potentially also changing the total surface reservoir size (48). Model simulations suggest that this could cause VOCs with higher log *K*_OA_ values to partition to a greater extent into the reservoirs while VOCs with a log *K*_OA_ < 4.4 would still not partition significantly. The partitioning and diffusion of VOCs into various materials and the effect of relative humidity as well as air and surface temperatures would need to be better understood for the model to be extrapolated to different furnished indoor environments (49). We also note that bulk diffusivity of different molecules may vary depending on the character of the molecule (18), affecting effective partitioning capacity. Additionally, the presence of people can influence the composition of indoor surfaces due to their activities and the deposition of skin flakes and oils (28).

Our estimation of the half-life ($t_{\frac{1}{2}}$) in surface reservoirs of 8 μm and 10 nm in thickness highlights the strong dependence of a compound’s indoor persistence on log *K*_OA_ and surface reservoir size. This analysis shows that, depending on the log *K*_OA_ value of the contaminant, the persistence of nonreactive species that are in equilibrium with large surface reservoirs will extend from short decay lifetimes that indicate instantaneous desorption, to timescales that rival the age of the building. In the latter case (roughly, log *K*_OA_ > 9) no degree of ventilation will lead to substantial loss of the contaminant.

Past partitioning models have had to rely on assumed sizes and composition of the partitioning reservoirs (4, 6, 35, 36, 50, 51). This work actually measures the partitioning capacity, demonstrating that contaminants with log *K*_OA_ values above 5 will be more strongly partitioned to surface reservoirs rather than to air. Thus, these findings may impact our understanding of how humans are exposed to organic contaminants with regard to inhalation exposure for high *K*_OA_ chemicals, relative to dermal uptake and nondietary exposure mechanisms which are directly related to indoor surface composition.

## Materials and Methods

### Experimental Design.

The *CASA* campaign took place from March 2 to April 11, 2022, at the National Institute of Standards and Technology’s (NIST) Net-Zero Energy Residential Test Facility (NZERTF), which undergoes air change with the outdoors largely via mechanical ventilation and to a smaller degree by infiltration (11, 29, 32, 52, 53). On separate occasions, short timescale injections (up to 6 min long) of two sets of VOCs into the KDL of the first floor of the house (Fig. 1*A*) occurred using a custom-designed mixture of relatively high volatility compounds referred to as the *CASA* cocktail and an insecticide spray, which contained lower volatility compounds. A portable fan was used to enhance VOC mixing within the KDL. Distribution of VOCs to the rest of the house occurred via the continuous operation of the ventilation system fan and heating/cooling system.

A proton-transfer-reaction mass spectrometer (PTR-MS, Tofwerk Inc.) (54) was used to measure VOC gas-phase mixing ratios. The PTR-MS was located in the garage, and measurements were made in the KDL for four hours after injection. VOCs within the insecticide spray were identified using thermal-desorption gas-chromatography mass spectrometry (TD-GC-MS). Additional details on the house operation, injection protocols, and PTR-MS and TD-GC-MS operation are in Text S1a-e.

### Kinetic Box Modeling with Surface Partitioning.

A schematic of the kinetic model used to describe the measurements is in Fig. 1*B*, and calculation details and equations are provided in the Supplementary Information (*SI Appendix*, Text S2). In brief, the NZERTF is modeled as a two-box system. This is required because mixing to the rest of the house is significantly slower than within the KDL. The mixing rates between the two regions of the house and with the outdoors are informed by detailed flow characterization of the house and by fitting to tracer decay observations. The gas-phase mixing ratios of VOCs in the KDL where the measurements were made were calculated using a series of coupled differential equations that express the time-dependent relationships between interzonal flow in the house, air change with the outdoors, deposition to surfaces within the house, and emissions. Note that a key parameter that controls the overall mass transfer rate of the VOCs to the house surfaces is the octanol-air partition coefficient, *K*_OA_. Mass transfer within the surface reservoir has been treated using both the α_eff_ method and the kinetic multilayer model with descriptions and comparisons of the *Materials and Methods* being provided in the *SI Appendix*.

## Disclaimer

Certain equipment, instruments, or materials, commercial or noncommercial, are identified in this paper to specify the experimental procedure adequately. Such identification is not intended to imply recommendation or endorsement of any product or service by NIST, nor is it intended to imply that the materials or equipment identified are necessarily the best available for the purpose. The policy of NIST is to use the International System of Units in all publications. In this document, however, some units are presented in the system prevalent in the relevant discipline.

## Supplementary Material

Appendix 01 (PDF)

## Data Availability

All study data are included in the article and/or *SI Appendix*. The data presented in the paper can be obtained in digital format from Borealis (55).

## References

[1] C. J. Weschler, W. W. Nazaroff, Svoc partitioning between the gas phase and settled dust indoors. Atmos. Environ. **44**, 3609–3620 (2010).

[2] B. C. Singer, A. T. Hodgson, K. S. Guevarra, E. L. Hawley, W. W. Nazaroff, Gas-phase organics in environmental tobacco smoke. 1. Effects of smoking rate, ventilation, and furnishing level on emission factors. Environ. Sci. Technol. **36**, 846–853 (2002). 11918006 10.1021/es011058w

[3] C. Wang , Surface reservoirs dominate dynamic gas-surface partitioning of many indoor air constituents. Sci. Adv. **6**, eaay8973 (2020). 32128415 10.1126/sciadv.aay8973 PMC7030931

[4] C. J. Weschler, W. W. Nazaroff, Semivolatile organic compounds in indoor environments. Atmos. Environ. **42**, 9018–9040 (2008).

[5] B. C. Singer, K. L. Revzan, T. Hotchi, A. T. Hodgson, N. J. Brown, Sorption of organic gases in a furnished room. Atmos. Environ. **38**, 2483–2494 (2004).

[6] L. Li, J. A. Arnot, F. Wania, How are humans exposed to organic chemicals released to indoor air? Environ. Sci. Technol. **53**, 11276–11284 (2019). 31496218 10.1021/acs.est.9b02036

[7] D. Won, R. L. Corsi, M. Rynes, Sorptive interactions between VOCs and indoor materials. Indoor Air **11**, 246–256 (2001). 11761600 10.1034/j.1600-0668.2001.110406.x

[8] R. B. Jørgensen, O. Bjørseth, Sorption behaviour of volatile organic compounds on material surfaces—The influence of combinations of compounds and materials compared to sorption of single compounds on single materials. Environ. Int. **25**, 17–27 (1999).

[9] R. B. Jørgensen, O. Bjørseth, Chamber testing of adsorption of volatile organic compounds (VOCs) on material surfaces. Indoor Air **9**, 2–9 (1999). 10195270 10.1111/j.1600-0668.1999.t01-3-00002.x

[10] X. Wang, Y. Zhang, J. Xiong, Correlation between the solid/air partition coefficient and liquid molar volume for VOCs in building materials. Atmos. Environ. **42**, 7768–7774 (2008).

[11] D. K. Farmer , The chemical assessment of surfaces and air (CASA) study: Using chemical and physical perturbations in a test house to investigate indoor processes. Environ. Sci. Process. Impacts **27**, 1551–1572 (2024). 10.1039/d4em00209a 38953218

[12] D. K. Farmer , Overview of HOMEChem: House observations of microbial and environmental chemistry. Environ Sci Process Impacts **21**, 1280–1300 (2019). 31328749 10.1039/c9em00228f

[13] Y. Liu , Characterizing sources and emissions of volatile organic compounds in a northern California residence using space- and time-resolved measurements. Indoor Air **29**, 630–644 (2019). 31004537 10.1111/ina.12562

[14] J. P. D. Abbatt , How should we define an indoor surface?. Indoor Air **32**, e12955 (2022). 35104002 10.1111/ina.12955

[15] Q.-T. Liu, R. Chen, B. E. McCarry, M. L. Diamond, B. Bahavar, Characterization of polar organic compounds in the organic film on indoor and outdoor glass windows. Environ. Sci. Technol. **37**, 2340–2349 (2003). 12831015 10.1021/es020848i

[16] C. Y. Lim, J. P. Abbatt, Chemical composition, spatial homogeneity, and growth of indoor surface films. Environ. Sci. Technol. **54**, 14372–14379 (2020). 33156609 10.1021/acs.est.0c04163

[17] C. J. Weschler, W. W. Nazaroff, Growth of organic films on indoor surfaces. Indoor Air **27**, 1101–1112 (2017). 28556424 10.1111/ina.12396

[18] L. B. Algrim, D. Pagonis, J. A. Gouw, J. L. Jimenez, P. J. Ziemann, Measurements and modeling of absorptive partitioning of volatile organic compounds to painted surfaces. Indoor Air **30**, 745–756 (2020). 32077147 10.1111/ina.12654

[19] F. Wania, D. MacKay, Peer reviewed: Tracking the distribution of persistent organic pollutants. Environ. Sci. Technol. **30**, 390A–396A (1996). 10.1021/es962399q 21649427

[20] H. Xiao, F. Wania, Is vapor pressure or the octanol–air partition coefficient a better descriptor of the partitioning between gas phase and organic matter?. Atmos. Environ. **37**, 2867–2878 (2003).

[21] A. Finizio, D. Mackay, T. Bidleman, T. Harner, Octanol-air partition coefficient as a predictor of partitioning of semi-volatile organic chemicals to aerosols. Atmos. Environ. **31**, 2289–2296 (1997).

[22] J. F. Pankow, Further discussion of the octanol/air partition coefficient Koa as a correlating parameter for gas/particle partitioning coefficients. Atmos. Environ. **32**, 1493–1497 (1998).

[23] X. Duan, Y. Zhu, J. Cao, An empirical approach for computing an effective whole-house surface partitioning coefficient of indoor organic pollutants. Build. Environ. **280**, 113097 (2025).

[24] J. Yu, F. Wania, J. P. D. Abbatt, A new approach to characterizing the partitioning of volatile organic compounds to cotton fabric. Environ. Sci. Technol. **56**, 3365–3374 (2022). 35230819 10.1021/acs.est.1c08239

[25] C. M. A. Eichler , Cloth-air partitioning of neutral per- and polyfluoroalkyl substances (PFAS) in North Carolina homes during the indoor PFAS assessment (IPA) campaign. Environ. Sci. Technol. **57**, 15173–15183 (2023). 37757488 10.1021/acs.est.3c04770 PMC11182342

[26] P. S. J. Lakey , Spatial and temporal scales of variability for indoor air constituents. Commun. Chem. **4**, 110 (2021). 36697551 10.1038/s42004-021-00548-5 PMC9814873

[27] C. Arata , Volatile organic compound emissions during HOMEChem. Indoor Air **31**, 2099–2117 (2021). 34272904 10.1111/ina.12906

[28] Y. Liu , Observing ozone chemistry in an occupied residence. Proc. Natl. Acad. Sci. **118**, e2018140118 (2021). 33526680 10.1073/pnas.2018140118 PMC8017968

[29] J. Li , The persistence of smoke VOCs indoors: Partitioning, surface cleaning, and air cleaning in a smoke-contaminated house. Sci. Adv. **9**, eadh8263 (2023). 37831770 10.1126/sciadv.adh8263 PMC10575580

[30] P. S. J. Lakey, B. E. Cummings, M. S. Waring, G. C. Morrison, M. Shiraiwa, Effective mass accommodation for partitioning of organic compounds into surface films with different viscosities. Environ. Sci.: Processes Impacts **25**, 1464–1478 (2023). 10.1039/d3em00213f 37560969

[31] P. S. J. Lakey, C. M. A. Eichler, C. Wang, J. C. Little, M. Shiraiwa, Kinetic multi-layer model of film formation, growth, and chemistry (KM-FILM): Boundary layer processes, multi-layer adsorption, bulk diffusion, and heterogeneous reactions. Indoor Air **31**, 2070–2083 (2021). 33991124 10.1111/ina.12854

[32] M. F. Link , Ventilation in a residential building brings outdoor NO x indoors with limited implications for VOC oxidation from NO 3 radicals. Environ. Sci. Technol. **57**, 16446–16455 (2023). 37856830 10.1021/acs.est.3c04816

[33] R. E. O’Brien , Emerging investigator series: Chemical and physical properties of organic mixtures on indoor surfaces during HOMEChem. Environ. Sci. Process. Impacts. **23**, 559–568 (2021). 33870396 10.1039/d1em00060h

[34] J. M. Silberstein , Residual impacts of a wildland urban interface fire on urban particulate matter and dust: A study from the Marshall Fire. Air Qual. Atmos. Health **16**, 1839–1850 (2023).

[35] W. D. Fahy, F. Wania, J. P. D. Abbatt, When does multiphase chemistry influence indoor chemical fate?. Environ. Sci. Technol. **58**, 4257–4267 (2024). 38380897 10.1021/acs.est.3c08751

[36] J. P. D. Abbatt, C. Wang, The atmospheric chemistry of indoor environments. Environ. Sci. Process. Impacts **22**, 25–48 (2020). 31712796 10.1039/c9em00386j

[37] G. C. Morrison, W. W. Nazaroff, The rate of ozone uptake on carpet: Mathematical modeling. Atmos. Environ. **36**, 1749–1756 (2002).

[38] B. C. McDonald , Volatile chemical products emerging as largest petrochemical source of urban organic emissions. Science **1979**, 760–764 (2018). 10.1126/science.aaq0524 29449485

[39] J. P. Winickoff , Beliefs about the health effects of “thirdhand” smoke and home smoking bans. Pediatrics **123**, e74–e79 (2009). 19117850 10.1542/peds.2008-2184 PMC3784302

[40] M. Sleiman , Formation of carcinogens indoors by surface-mediated reactions of nicotine with nitrous acid, leading to potential thirdhand smoke hazards. Proc. Natl. Acad. Sci. **107**, 6576–6581 (2010). 20142504 10.1073/pnas.0912820107 PMC2872399

[41] P. F. DeCarlo, A. M. Avery, M. S. Waring, Thirdhand smoke uptake to aerosol particles in the indoor environment. Sci. Adv. **4**, eaap8368 (2018). 29750194 10.1126/sciadv.aap8368 PMC5942907

[42] Z. Zhou , Multiphase ozonolysis of oleic acid-based lipids: Quantitation of major products and kinetic multilayer modeling. Environ. Sci. Technol. **56**, 7716–7728 (2022). 35671499 10.1021/acs.est.2c01163

[43] S. Zhou , Multiphase reactivity of polycyclic aromatic hydrocarbons is driven by phase separation and diffusion limitations. Proc. Natl. Acad. Sci. **116**, 11658–11663 (2019). 31142653 10.1073/pnas.1902517116 PMC6575172

[44] C.-G. Bornehag , The association between asthma and allergic symptoms in children and phthalates in house dust: A nested case-control study. Environ. Health Perspect. **112**, 1393–1397 (2004). 15471731 10.1289/ehp.7187 PMC1247566

[45] T. Vasiljevic, T. Harner, Bisphenol A and its analogues in outdoor and indoor air: Properties, sources and global levels. Sci. Total Environ. **789**, 148013 (2021). 34323825 10.1016/j.scitotenv.2021.148013

[46] S. M. Duncan , Dynamics of residential water-soluble organic gases: Insights into sources and sinks. Environ. Sci. Technol. **53**, 1812–1821 (2019). 30633495 10.1021/acs.est.8b05852 PMC7279883

[47] Y. Fang , A molecular picture of surface interactions of organic compounds on prevalent indoor surfaces: Limonene adsorption on SiO2. Chem. Sci. **10**, 2906–2914 (2019). 30996868 10.1039/c8sc05560b PMC6428143

[48] A. Manuja , Total surface area in indoor environments. Environ. Sci. Process. Impacts **21**, 1384–1392 (2019). 31246204 10.1039/c9em00157c

[49] Y. Zhang, J. Xiong, J. Mo, M. Gong, J. Cao, Understanding and controlling airborne organic compounds in the indoor environment: Mass transfer analysis and applications. Indoor Air **26**, 39–60 (2016). 25740682 10.1111/ina.12198

[50] X. Zhang, M. L. Diamond, C. Ibarra, S. Harrad, Multimedia modeling of polybrominated diphenyl ether emissions and fate indoors. Environ. Sci. Technol. **43**, 2845–2850 (2009). 19475960 10.1021/es802172a

[51] D. H. Bennett, E. J. Furtaw, Fugacity-based indoor residential pesticide fate model. Environ. Sci. Technol. **38**, 2142–2152 (2004). 15112818 10.1021/es034287m

[52] D. G. Poppendieck, L. C. Ng, A. K. Persily, A. T. Hodgson, Long term air quality monitoring in a net-zero energy residence designed with low emitting interior products. Build. Environ. **94**, 33–42 (2015).

[53] D. Poppendieck, M. Gong, S. Zimmerman, L. Ng, Evaluation of a four-zone indoor exposure model for predicting TCPP concentrations in a low-energy test house. Build. Environ. **199**, 107888 (2021). 10.1016/j.buildenv.2021.107888 PMC10947393 38500674

[54] J. Krechmer , Evaluation of a new reagent-ion source and focusing ion-molecule reactor for use in proton-transfer-reaction mass spectrometry. Anal. Chem. **90**, 12011–12018 (2018). 30220198 10.1021/acs.analchem.8b02641

[55] J. Abbatt, Data for PNAS – Yu – 2025. Borealis. 10.5683/SP3/G50UKG. Deposited 29 August 2025.

