# Supplementary material for: VOC injection into a house reveals large surface reservoir sizes in an indoor environment
Source: Proc Natl Acad Sci U S A. 2025 Sep 22;122(39):e2503399122. doi: 10.1073/pnas.2503399122 (PMC12501166; doi:10.1073/pnas.2503399122)
Supplement: Supplementary file 1 — Appendix 01 (PDF) [file pnas.2503399122.sapp.pdf]

PNAS

# Supporting Information for

VOC Injection into a House Reveals Large Surface Reservoir Sizes in an Indoor Environment

Jie Yu,[1,#] Pascale S. J. Lakey,[2,#] Jenna C. Ditto,[3] Han N. Huynh,[4,5] Michael F. Link,[6] Dustin Poppendieck,[6] Stephen M. Zimmerman,[6] Xing Wang,[7] Delphine K. Farmer,[8] Marina E. Vance,[9] Jonathan P.D. Abbatt[1,*], Manabu Shiraiwa[2,*]

[1] Department of Chemistry, University of Toronto, 80 St. George Street, Toronto, Ontario, M5S 3H6, Canada

[2] Department of Chemistry, University of California, Irvine, California 92697-2025, United States

[3] Department of Energy, Environmental, and Chemical Engineering, Washington University in St. Louis, St. Louis, Missouri 63130, United States

[4] Cooperative Institute for Research in Environmental Sciences, University of Colorado, Boulder, Colorado 80309, United States

[5] NOAA Chemical Sciences Laboratory, Boulder, Colorado 80309, United States

[6] National Institute of Standards and Technology, Gaithersburg 20899, Maryland, United States

[7] Department of Chemical Engineering and Applied Chemistry, University of Toronto, Toronto, Ontario M5S 3E5, Canada

[8] Department of Chemistry, Colorado State University, Fort Collins, Colorado 80523, United States

[9] Department of Mechanical Engineering, University of Colorado, Boulder, Colorado 80309, United States

* Corresponding Authors: Manabu Shiraiwa, Jonathan P.D. Abbatt

**Email:** m.shiraiwa@uci.edu, jonathan.abbatt@utoronto.ca

**This PDF file includes:**

Text S1 to S5
Figures S1 to S16
Tables S1 to S5
SI References

**Supporting Information**

**Text S1. Additional information on house operation, injection protocols and instrumentation.**

**(a) House Operation at the Net Zero Energy Residential Test Facility (NZERTF)**

The insecticide spray addition (March 10) and the cocktail injection (March 31) were both conducted on the 1$^{st}$ floor of the house (Figure 1a) where the 24-hour average temperature was 24 ± 1 °C, and the 24-hour average relative humidity (RH) was 25 ± 1% and 53 ± 4% for March 10 and March 31, respectively. To enhance mixing in the house, the fan for the heat pump operated continuously, returning air to the unit from the 1$^{st}$ and 2$^{nd}$ floors and supplying air to all three levels. This maximum-setting flow rate was 1150 $m^3$ $h^{-1}$ ± 30 $m^3$ $h^{-1}$. As well, fans were used in the open area of the 1$^{st}$ floor area to enhance VOC mixing rates at that location. The air change rate (ACR) of the NZERTF is driven by both infiltration and mechanical ventilation (Table S1). The heat-recovery ventilation system (HRV) supplies outdoor air indoors through the ducts on the 1$^{st}$ and 2$^{nd}$ floors at a fixed airflow rate (Table S1). The average whole house ACR during the campaign, measured by decay of sulfur hexafluoride, was ≈ 0.24 $h^{-1}$, with values on March 10 and March 31 of 0.25 and 0.24 $h^{-1}$, respectively.

**(b) Insecticide Spray Addition**

An insecticide aerosol spray can was purchased from a local retail store in Gaithersburg, MD, USA. House access was not allowed 10 minutes prior to the addition event and not until 4 hours after. A fan, placed at the southwest corner of the living room and facing northeast, was turned on remotely 3 minutes prior to the addition. At the same time, a participant entered the house to spray the insecticide around the living room area (dashed box in Figure 1a) for 25 seconds according to instructions on the can and exited the house immediately. The fan was turned off after 4 minutes after the end of the spray. 44 g of the spray can contents were used. A 30-minute $O_3$ addition was performed through the HRV system 1 hour after the spray, to reach a peak level of 50 ppb on the 1$^{st}$ floor, with 3 ppb present before addition. Ozone ($O_3$) was produced by supplying ultra-high purity $O_2$ to an $O_3$ generator. Note we express mixing ratios in this paper in parts-per notation defined as the number of moles of an analyte per mole of air. A second insecticide addition was performed 4 hours after the first but not used for this analysis due to the growing uncertainty in the house background levels many hours after the background measurement period.

**(c) CASA Cocktail Injection**

Known identities of chemicals including acetone, toluene-$d_8$, o-xylene, chlorobenzene, 2-pentanone, 2-heptanone, α-pinene, 1-hexene, isoprene and furfural were purchased from Sigma Aldrich Co. (St. Louis, MO, USA) and used for *CASA* cocktail injection.

The injection setup was installed in the porch (Figure 1a), isolated from the main house. No house access was allowed 1 hour before and 10 hours after the injection. A fan was placed 0.4 m from the north kitchen/dining room wall (Figure 1a) facing the dining room and turned on remotely 3 minutes before the injection. 0.05 mL of each cocktail compound was added to a 1 L Erlenmeyer flask immediately prior to the injection. The flask was capped with a sealed fritted glass bubbler insert but it is possible that some of the more volatile species were lost from the flask as the mixture was prepared. The injection started when zero air was supplied to the flask through the bubbler insert at 10 L $min^{-1}$, while quickly submerging the flask into a 90 °C water bath. The other end of the bubbler was connected to tubing (1.27 cm OD and 0.95 cm ID) that passed through the door (1 m above the floor, positioned 1.1 m inside the house and pointed perpendicular to the north kitchen/dining room wall) to deliver evaporated chemicals to the dining room. The injection lasted for 6 minutes until the chemicals were fully evaporated. The fan was turned off 2 minutes after the injection and the tubing was removed from the port 5 minutes after the injection. Cocktail injections earlier in the campaign were not analyzed because an order of magnitude more material was injected on those occasions. This led to longer and variable total injection times for the different compounds, which are harder to model.

**(d) Gas-phase Measurements by PTR-MS and TD-GC-MS**

The VOC gas-phase mixing ratios were measured by a proton-transfer-reaction mass spectrometer (PTR-MS, Tofwerk Inc.) (1). From the garage, it sampled the main house air via 30.5 m long Teflon tubing (1.27 cm OD and 0.95 cm ID) held at 50 °C. The sampling inlet was placed in the dining room (Figure 1a), 1.6 m above the floor and 2 m from the nearest wall. A Teflon particle filter in a PFA (Perfluoroalkoxy) holder was placed before the sampling inlet. 4 L $min^{-1}$ flow was pulled from the dining room and the PTR-MS sampled 100 mL $min^{-1}$ from the main flow. The time-series for the first 4 hours immediately after addition was used. A constant house background signal, averaged from the last 60 minutes before each addition, was subtracted from the raw signal. Ion signals that were less than 10 ions $s^{-1}$ were removed. Additional details on PTR-MS operation and data processing are in Text S1e. The PTR-MS has been demonstrated to have very high linearity in its response.(1)

VOCs of known identities were injected into the house during the *CASA* cocktail experiment, thus the corresponding parent ion signals, $[M+H]^+$, were monitored by PTR-MS. By contrast, the content of the insecticide spray can is unknown. As a result, in addition to identifying commonly observed ions via comparison to prior PTR-MS analyses, a post-campaign gas-phase composition

analysis was conducted using a thermal-desorption (TDS-3, Gerstel) gas-chromatography (7890B, Agilent) mass-spectrometry (5977A, Agilent) system (TD-GC-MS) in electron impact ionization mode. For the insecticide spray nine chemicals were identified based on combined PTR-MS and TD-GC-MS analyses. Detailed information on the spray set-up, TD-GC-MS operation method, and chemical identification is provided in Text S1(f).

**(e) Vocus PTR-MS operation and data processing**

The PTR-MS operating conditions were as follows:

(1) the reagent ions ($H_3O^+$) were produced in the positively charged ion source originating from HPLC-grade milli-Q water at a flow rate of 20 *m*L min$^{-1}$;

(2) the focusing ion-molecule reactor (FIMR) was operated at a temperature of 100 °C, IMR front voltage of 600 V, IMR back voltage of 30 V, a pressure of 2.2 mbar, a discharge voltage of 430 V and a discharge current of 2 mA. The reduced electric field ratio (E/N) was 130 Td;

(3) the mass resolution during the campaign was approximately 9000 at mass-to-charge (m/z) 200;

(4) the recorded m/z range was 3-499. The time resolution for data averaging was set at 4 seconds.

Tofware (version 3.2.5) was used for post-campaign data processing. Mass calibration was first performed to the raw data file, in which the mean peak fitting residual < 5 ppm was obtained for each mass calibration ion.

For the cocktail injection experiment, given that the identity of the injected chemicals is known, high-resolution peak fitting for each parent ion $[M+H]^+$ was applied to construct a peak list for the injected chemicals, except for toluene-d8 ($C_7D_8H^+$). Note that due to deuterium/hydrogen (D/H) exchange reactions with $H_3O^+$ in the drift tube (2), $C_7D_3H_6^+$ is the ion that showed the strongest signal for toluene-d8, and thus was used for time series analysis.

A non-targeted PTR-MS analysis was applied for the measurements of the insecticide spray can; details are included in Text S1(f).

**(f) Identity assignment method for the insecticide spray using PTR-MS and TD-GC-MS**

For the insecticide spray addition experiment, a non-targeted analysis was applied, initiated in Tofware by the following steps:

(1) scan through the time series of ions (m/z 3-499) at unit mass resolution, identifying the unit mass m/z that showed an increase in signal upon spraying;

(2) perform a high-resolution peak assignment for each of these unit masses, assign a formula to the peak that showed an increase, with a mass uncertainty < 3 ppm;

(3) assign a chemical identity according to the formula based on availability in the PTR library provided by Pagonis et al (2019) (3). Note that here we assume these ions represent the parent ion. The presence of other fragments of bigger molecules cannot be eliminated, despite PTR being a relatively soft ionization technique.(1)

As a result, a primary compound list was generated.

Subsequent construction of a secondary compound list was based on a post-campaign TD-GC-MS analysis of the insecticide spray can contents. A small amount (one gentle press on the nozzle, less than 1 second) of insecticide was sprayed into a 0.6 m × 0.6 m × 0.5 m acrylic box (pre-cleaned by methanol), and gas-phase compounds were collected onto a Tenax TA Sorbent tube (P09725, Gerstel) by pulling a 100 *m*L min$^{-1}$ flow by an external pump for 1 minute. A Teflon filter in a PFA filter holder was placed between the box and the Tenax tube for particle removal. A blank sample was also collected under the same conditions when nothing was sprayed into the box.

The two Tenax tubes were then individually analyzed by a TD-GC-MS system, with the operation method adapted from Wang and Chan (2023) (4). The thermal desorption system (TDS3, Gerstel) was initially set to 50 °C, with a temperature ramp of 60 °C min$^{-1}$ to reach 320 °C and then held for 4 minutes. Desorbed analytes were sent through a heated transfer line (300 °C) by helium gas to a cooled injection system (CIS) set at −40 °C until the desorption was complete. The CIS was then heated at 12 °C s$^{-1}$ to reach 320 °C and held for 5 minutes. Analytes were delivered to a low-bleed GC column (Rtx-5MS, 30 m, 0.25 mm ID, 1 µm film thickness) in the GC oven set at 50 °C, heated at 7 °C min$^{-1}$ to reach 320 °C, then held for 7.5 minutes. The electron impact ionization system was set at +70 eV using a standard tungsten filament with a source temperature of 230 °C. The MS was operated at 3.1 Hz with an m/z acquisition range of 35-550.

For GC-MS data processing, the Agilent Mass Hunter software was used to identify chromatographic peaks by matching the mass spectral peak features with the NIST08 mass spectral library after background subtraction. If the matching percentage is higher than 65%, then the assigned chemical was added to the secondary compound list. The corresponding PTR-MS dataset collected for the insecticide spray was scanned again by performing a high-resolution peak assignment based on the identity provided by the NIST library. Only the chemicals that also showed

PTR-MS response upon spraying remained in the secondary compound list. The presence of other structural isomers cannot be eliminated.

Lastly, if a chemical in the primary compound list was not included in the secondary compound list, a set of isomers of that chemical, as provided in the NIST Chemical WebBook was selected (5), and their log $K_{OA}$ values were obtained by polyparameter linear free energy relationship (ppLFER) prediction in the UFZ-LSER database (6). As seen from Table S4, the log $K_{OA}$ values of isomers do not differ greatly for most of the compounds identified. As a result, a combined list of identified compounds can be finalized and is provided in Table S5.

**Text S2. Additional method details on kinetic box modeling with surface partitioning**

A schematic of the kinetic model is in Figure 1b with relevant calculations described below. The gas-phase mixing ratios of compounds in the open area where measurements were performed are calculated using a series of interlinked coupled, differential equations (E1 and E2). In particular, the average gas-phase concentration of compound X in the KDL ($[\mathrm{X}]_{\mathrm{g,KDL}}$, in $\mathrm{m}^{-3}$) is determined as follows (7, 8):

$$\frac{d[\mathrm{X}]_{\mathrm{g,KDL}}}{dt} = v_{\mathrm{d,KDL,X}}\left(\frac{[\mathrm{X}]_{\mathrm{abs,KDL}}}{L_{\mathrm{KDL}}K_{\mathrm{OA,X}}} - [\mathrm{X}]_{\mathrm{g,KDL}}\right)\frac{A_{\mathrm{KDL}}}{V_{\mathrm{KDL}}} - k_{\mathrm{KDL,out}}[\mathrm{X}]_{\mathrm{g,KDL}} + E_{\mathrm{KDL,X}} + k_{\mathrm{KDL,in}}[\mathrm{X}]_{\mathrm{g,H}} \quad \text{(E1)}$$

where $u_{\mathrm{d,KDL,X}}$ ($\mathrm{m\ s^{-1}}$) is the deposition velocity of compound X to surfaces in the KDL, $L_{\mathrm{KDL}}$ (m) is the octanol-equivalent average thickness of surface films and reservoirs in the KDL. We do not consider interactions with particles given their very low partitioning capacity relative to surface reservoirs. We also assume that the mixing ratios of compounds outside the house are negligible. $L_{\mathrm{KDL}}$ is estimated by fitting to measurements. $K_{\mathrm{OA,X}}$ is the octanol-air partitioning coefficient of compound X, which is the ratio of the equilibrium concentration of X in octanol (e.g., in surfaces) to its concentration in air. Log $K_{\mathrm{OA,X}}$ values were obtained by linear free energy relationships from an online database(6). $A_{\mathrm{KDL}}$ ($\mathrm{m}^2$) and $V_{\mathrm{KDL}}$ ($\mathrm{m}^3$) are the surface area and volume of the KDL, respectively. $E_{\mathrm{KDL,X}}$ ($\mathrm{m^{-3}\ s^{-1}}$) is the emission rate of compound X in the KDL, a constant emission rate was assumed for both addition events for the entire measurement period as described previously. $k_{\mathrm{KDL,out}}$ ($\mathrm{s}^{-1}$) and $k_{\mathrm{KDL,in}}$ ($\mathrm{s}^{-1}$) are the first-order mass-transport rates of species X out of and into the KDL, respectively. $k_{\mathrm{KDL,out}}$ is calculated as ($Q_{\mathrm{KDL,H}}$ + $Q_{\mathrm{exf,KDL,out}}$)/ $V_{\mathrm{KDL}}$ and $k_{\mathrm{KDL,in}}$ is calculated as $Q_{\mathrm{H,KDL}}$/ $V_{\mathrm{KDL}}$, where $Q$ denotes volumetric flow rates shown in Figure 1b. $[\mathrm{X}]_{\mathrm{abs,KDL}}$ ($\mathrm{m}^{-2}$) is the absorbed concentration per surface area in the KDL and is calculated using the following equation (7, 8):

$$\frac{d[\mathrm{X}]_{\mathrm{abs,KDL}}}{dt} = v_{\mathrm{d,KDL,X}}\left([\mathrm{X}]_{\mathrm{g,KDL}} - \frac{[\mathrm{X}]_{\mathrm{abs,KDL}}}{L_{\mathrm{KDL}}K_{\mathrm{OA,X}}}\right) \quad \text{(E2)}$$

$\frac{[\mathrm{X}]_{\mathrm{abs,KDL}}}{L_{\mathrm{KDL}}K_{\mathrm{OA,X}}}$ represents gas-phase concentration of X right above the surface and the right-hand side of equation E2 represents mass transfer driven by concentration gradients(9). The average gas-

phase concentration of compound X in the rest of the house ($[X]_{g,H}$, in $m^{-3}$) was calculated using the model as follows (7, 8):

$$\frac{d[\mathrm{X}]_{\mathrm{g,H}}}{dt} = v_{\mathrm{d,H,X}}\left(\frac{[\mathrm{X}]_{\mathrm{abs,H}}}{L_{\mathrm{H}}K_{\mathrm{OA,X}}} - [\mathrm{X}]_{\mathrm{g,H}}\right)\frac{A_{\mathrm{H}}}{V_{\mathrm{H}}} - k_{\mathrm{H,out}}[\mathrm{X}]_{\mathrm{g,H}} + k_{\mathrm{H,in}}[\mathrm{X}]_{\mathrm{g,KDL}} - k_{loss}[\mathrm{X}]_{\mathrm{g,H}} \quad \text{(E3)}$$

where $u_{d,H,X}$ (m $s^{-1}$) is the deposition velocity of compound X to surfaces in the rest of the house, $L_H$ (m) is the octanol-equivalent average thickness of surface films and reservoirs in the rest of the house, and $A_H$ ($m^2$) and $V_H$ ($m^3$) are the surface and volume of the rest of the house, respectively. $k_{H,out}$ ($s^{-1}$) and $k_{H,in}$ ($s^{-1}$) are the first-order mass-transport rates of X out of and into the rest of the house, respectively. $k_{H,out}$ is calculated as ($Q_{H,KDL}$ + $Q_{exf,H,out}$ + $Q_{HRV,H,out}$)/ $V_H$ and $k_{H,in}$ is calculated as $Q_{KDL,H}$/ $V_H$. $k_{loss}$ is a first-order loss process which is possibly a transport-limited sorption loss affecting all VOCs equally and is determined by fitting to measurements. We assume that the transport-limited process occurs only in the rest of the house and the $k_{loss}$ term therefore does not appear in Equation E1. Note that $k_{loss}$ has not been included in the $t_{\frac{1}{2},surf}$ calculations due to uncertainty with regards to its origin and further experiments are required to determine the nature of this loss. If it represents a loss to a deep reservoir from which VOCs can repartition over long timescales, $t_{\frac{1}{2},surf}$ could increase.

The concentrations and model fits in the rest of the house are unknown. $[X]_{abs,H}$ ($m^{-2}$) is the absorbed concentration per surface area in the rest of the house and is calculated using the following equation (7, 8):

$$\frac{d[\mathrm{X}]_{\mathrm{abs,H}}}{dt} = v_{\mathrm{d,H,X}}\left([\mathrm{X}]_{\mathrm{g,H}} - \frac{[\mathrm{X}]_{\mathrm{abs,H}}}{L_{\mathrm{H}}K_{\mathrm{OA,X}}}\right) \quad \text{(E4)}$$

The deposition velocities in the equations above can be calculated to be in the range of 2.3 – 4.7 m $h^{-1}$ using equation E5 (7):

$$v_{\mathrm{d,X}} = \left(\frac{1}{h_{\mathrm{m}}} + \frac{1}{\alpha_{\mathrm{eff,X}}\omega_{\mathrm{X}}/4}\right)^{-1} \quad \text{(E5)}$$

where $h_m$ (m) is the convective mass transfer coefficient which has been set to 3 m $h^{-1}$ for consistency with the literature (8). $\omega_X$ (m $s^{-1}$) is the mean thermal velocity of compound X. $\alpha_{eff,X}$ is the effective mass accommodation coefficient of compound X, which is defined as the probability of a molecule colliding with a surface being incorporated into the condensed phase, as determined as a function of the surface accommodation coefficient ($\alpha_s$), $K_{OA}$ and bulk diffusion coefficient ($D_b$, in $m^2$ $s^{-1}$) using the following equation (7):

$$\alpha_{\mathrm{eff}} = \alpha_{\mathrm{s}}\frac{1}{1 + \frac{\alpha_{\mathrm{s}}\omega L}{12D_{\mathrm{b}}K_{OA}}} \quad \text{(E6)}$$

The application of $\alpha_{eff,X}$ allows us to consider the effect of potential kinetic limitations of bulk diffusion on partitioning if surface films adopt a viscous phase state. Note that for the base case simulations we assume $\alpha_{eff,X}$ = 1 without bulk diffusion limitations. For sensitivity tests where $\alpha_{eff,X}$ <

1, the diffusion limitation should be regarded as an average for the different types of surface materials in the KDL or rest of the house. Parameters used in Equations E1-6 are summarized in Table S1 and S2 and are either based on measurements, typical literature values, or by fitting to measurements. These are assumed to remain constant over the time interval of the experiment. Note that an assumption of non-reactive uptake has been made in this work. An assumption of well-mixed zones is also made, which prevents comparisons being made with measurements in the first 30 minutes after injection. Temperature gradients in the surface reservoirs, and associated variations on partitioning coefficient and bulk diffusivity, are uncertain and not treated in the model. The impact of surface partitioning on spatial and temporal scales of gas-phase chemicals has also been investigated, with methods described in Text S3a.

Note that the $\alpha_{eff}$ method is a simplification of a kinetic multi-layer model (10). The kinetic multi-layer model explicitly treats all relevant and advanced mass transport processes but are often too computationally expensive, while the $\alpha_{eff}$ method provides simple but effective approach to modify the surface deposition term. The development of the $\alpha_{eff}$ method is summarized in Figure S4. Figure S4a shows the treatment of an indoor space in our model with the different types of mass transport that are occurring. Figure S4b is a detailed schematic of our kinetic multi-layer model showing mass transport between individual layers. This treatment of bulk diffusion yields practically the same results (concentration profiles) as the solving of partial differential equation of the Fickian diffusion, but it is more flexible and requires no assumptions about interfacial transport (11, 12). Figure S4c shows how this detailed and explicit treatment can be simplified in the $\alpha_{eff}$ method by using an effective penetration depth to account for diffusion limitations. Further details can be found in Lakey et al. (2023) (7).

The kinetic multi-layer model treats flows into and out of the indoor spaces ($k_{out}$ and $k_{in}$), convective mass transfer from the air to the reservoir surface ($h_m$), the reversible partitioning fluxes between the near-surface gas phase and the first bulk reservoir layer ($J_{gs,b1}$ and $J_{b1,gs}$) and the fluxes between bulk reservoir layers (e.g. $J_{b1,b2}$ represents the flux from layer 1 to layer 2). The ordinary differential equations used in this version of the model are summarized below:

Gas phase:

$$\frac{d[\mathrm{X}]_\mathrm{g}}{dt} = -k_\mathrm{out}[\mathrm{X}]_\mathrm{g} + E_\mathrm{X} + k_\mathrm{in}[\mathrm{X}]_\mathrm{g} + h_\mathrm{m}([\mathrm{X}]_\mathrm{gs} - [\mathrm{X}]_\mathrm{g})\frac{A_S}{V_S} \quad \text{(E7)}$$

Near surface gas phase:

$$\frac{d[\mathrm{X}]_\mathrm{gs}}{dt} = h_\mathrm{m}\left([\mathrm{X}]_\mathrm{g} - [\mathrm{X}]_\mathrm{gs}\right)\frac{A_{mfp}}{V_{mfp}} + (J_{b1,gs} - J_{gs,b1})\frac{A_{mfp}}{V_{mfp}} \quad \text{(E8)}$$

First bulk layer:

$$\frac{d[\mathrm{X}]_\mathrm{b1}}{dt} = \left(J_\mathrm{gs,b1} - J_\mathrm{b1,b2}\right)\frac{A_{b1}}{V_{b1}} + \left(J_\mathrm{b2,b1} - J_\mathrm{b1,b2}\right)\frac{A_\mathrm{b1}}{V_\mathrm{b1}} \quad \text{(E9)}$$

Bulk layer k:

$$\frac{d[\mathrm{X}]_{\mathrm{b}k}}{dt} = \left(J_{bk-1,bk} - J_{bk,bk-1}\right)\frac{A_k}{V_k} + \left(J_{bk+1,bk} - J_{bk,bk+1}\right)\frac{A_{bk}}{V_{bk}} \quad \text{(E10)}$$

where $[X]_g$, $[X]_{gs}$, $[X]_{b1}$ and $[X]_k$ are the concentrations of X in the gas phase, near-surface gas phase, first bulk layer and layer k of the bulk, respectively. $A_S/V_S$, $A_{mfp}/V_{mfp}$, $A_{b1}/V_{b1}$ and $A_{bk}/V_{bk}$ are

the surface to volume ratios of the indoor space, the mean free path next to the surface, the first bulk layer and bulk layer k, respectively. Note that extensive testing has previously shown that there is good agreement between the $\alpha_{eff}$ method and multi-layer models when quasi-equilibrium has been reached in the penetration depth (7, 9). We did not resolve surface concentrations of adsorbed moelcules, which are not essential to quantify overall uptake into surface reservoirs in this study. The model also does not consider multilayer adsorption, which is unlikely for compounds with relatively high volatilities and at the low concentrations applied in this study. Note that high $K_{OA}$ and low volatile compounds, such as phthalates, may undergo multi-layer adsorption (10), which could lead to a greater effective partitioning coefficient into a surface reservoir. This aspect is beyond the scope of this study but should be investigated in a future study.

**Text S3. (a) Methods and (b) discussion on modeling the spatial and temporal scales of gas-phase chemicals.**

**(a) Methods**

Indoor air composition is highly dynamic as human activities occur and thus chemical species may be introduced or removed over a wide range of spatial and temporal scales (13). The potential heterogeneous distribution of chemicals emitted indoors can be modeled in the following manner.

The effective uptake coefficient of compound X ($\gamma_{eff,X}$) onto surfaces is calculated as follows:

$$\gamma_{\mathrm{eff,X}} = \frac{J_{\mathrm{ads,X}} - J_{\mathrm{des,X}}}{J_{\mathrm{coll,X}}} = \frac{v_{\mathrm{d,X}}\left([\mathrm{X}]_{\mathrm{g}} - \frac{[\mathrm{X}]_{\mathrm{abs}}}{LK_{\mathrm{OA,X}}}\right)}{\alpha_{\mathrm{s,X}}[\mathrm{X}]_{\mathrm{g}}\omega_{\mathrm{X}}/4} \quad \text{(E11)}$$

where $J_{ads,X}$, $J_{des,X}$ and $J_{coll,X}$ are the adsorption, desorption and collision fluxes ($m^{-2}$ $s^{-1}$) at the surface, respectively. The effective first-order decay rate of compound X ($k_{eff,X}$, in $s^{-1}$) can be determined using equation E8:

$$k_{\mathrm{eff,X}} = \frac{\gamma_{\mathrm{eff,X}}\ \omega_X A}{4V} \quad \text{(E12)}$$

As a way to express the temporal variation of chemicals, the half-life of compound X due to loss processes from a given area ($t_{\frac{1}{2},\mathrm{X}}$, in s) is calculated for the KDL and the rest of the house as (13):

$$t_{\frac{1}{2},\mathrm{KDL,X}} = \frac{\ln 2}{k_{\mathrm{eff,KDL,X}} + k_{\mathrm{KDL,out}}} \quad \text{(E13)}$$

$$t_{\frac{1}{2},\mathrm{H,X}} = \frac{\ln 2}{k_{\mathrm{eff,H,X}} + k_{\mathrm{H,out}} + k_{loss}} \quad \text{(E14)}$$

If the $k_{eff,X}$ value in equation E12 is negative due to net emission, then $k_{eff,X}$ is set to zero in Equation E13 and E14. As only loss terms are included in the equations, the calculated half-life will

not be fully representative of the true half-life in a space, but it is still useful for comparing the effect of different log $K_{OA}$ values on the uptake of compounds to surfaces and how this can impact the spatial scales of compounds. The spatial scale is represented by the transport distance ($d_t$, in m) for the corresponding $t_{\frac{1}{2}}$, which is determined using Equation E15:

$$d_t = \bar{\upsilon}\; t_{\frac{1}{2}} \qquad \text{(E15)}$$

where $\bar{\upsilon}$ is the average air velocity magnitude which was set to 0.03 m $s^{-1}$ for the purposes of this calculation but may be higher due to mixing fans in the KDL and HVAC circulation (13). A higher $\bar{\upsilon}$ would cause increased transport distances but would not impact the trend in the results.

**(b) Discussion: The Spatial and Temporal Gradients**

The time evolution of the average transport distance ($d_t$) that compounds will travel after a short-lived input pulse, and which may indicate the presence of gas-phase mixing ratio gradients indoors, was estimated as shown in Figure S12. The initial transport distance is controlled by deposition to the surface, which is limited by mass transport across the boundary layer (14). Subsequently the deposition to surfaces will affect the transport distance but as the net flux to the surface reduces the transport distance increases. Eventually the transport distance becomes constant and is controlled by air-exchange and transport between rooms in the house. This occurs when deposition to surfaces becomes low and when surfaces become net emitters (15). Compounds with higher log $K_{OA}$ values and larger reservoirs will lead to more deposition to films over a longer period and therefore the transport distance ($d_t$) remains lower. Note that these travel distances will also be influenced by internal mixing within zones which takes 5-15 minutes to be achieved and that this is not accounted for in the calculations.

Figure S13 summarizes the temporal and spatial scales of the different compounds 5 minutes and 1 hour after the start of the experiments. In the KDL these scales vary for the different compounds after 5 minutes depending on their log $K_{OA}$ values. As shown in Figure S12, in the KDL after 1 hour all compounds have reached equilibrium with the surface reservoirs (i.e. $\gamma_{eff,X}$ = 0) and they are no longer impacted by deposition to surfaces, thus leading to all compounds having the same travel distance ($d_t$) and half-life ($t_{\frac{1}{2}}$). In contrast, in the rest of the house after 1 hour compounds with the highest log $K_{OA}$ values do not reach equilibrium with the larger surface reservoirs and surface deposition continues to impact their temporal and spatial scales. Overall, we can conclude that when surface reservoirs are large, compounds with higher log $K_{OA}$ values possess a shorter travel distance and half-life due to stronger gas-surface interactions, and the mixing ratio gradient in the house exists for longer times before all compounds reach equilibrium.

**Text S4. Details on post-campaign $CO_2$ addition and measurement.**

A $CO_2$ injection was performed post-campaign at various injection locations during CASA, including the cocktail injection point. A $CO_2$ monitor was placed in the garage, connected to a sampling line in the living room. House settings were set to be identical as during the campaign (ventilation rate at 250 $m^3\ h^{-1}$ ± 20 $m^3\ h^{-1}$ and temperature at 24 °C). A high flow of 50 L $min^{-1}$ $CO_2$ was supplied from a cylinder (20% in Nitrogen) stored on the porch for 30 minutes. A fan was turned on 17 minutes after the start of the injection and kept on until the end of the injection. As a result, the air change rate (*ACR*) can be obtained from the $CO_2$ measurement (Figure S3).

**Text S5. Sample calculation of the $O_3$ reaction contribution to the overall decay.**

As the gas-phase rate constants for the insecticide compounds with $O_3$ are not available, a range of the gas-phase rate constants for a few common aromatic hydrocarbons are used here to demonstrate the minor contribution from $O_3$ reaction to the overall decay. Aromatics are chosen because of their known reactivity with $O_3$.

The range of the literature rate constants ($k_{O_3}$) of the gas-phase bimolecular reaction with $O_3$ for benzene (16), toluene (16), *m*-cresol (17), 1,2-dihydroxybenzene(18) and 1,2-dihydroxy-3-methylbenzene(18) at 298 K is 7 ×$10^{-23}$−2.8 ×$10^{-17}$ $cm^3$ $molecules^{-1}$ $s^{-1}$.

In the extreme case, when the level of $O_3$ was at a maximum 50 ppb, the maximum and minimum pseudo-rate constants of $k'_{O_3}$ can be calculated as:

$k'_{O_3} = k_{O_3} \times [O_3] = 7 \times 10^{-23}\ cm^3\ molecules^{-1}\ s^{-1} \times 50 \times (2.46 \times 10^{10}\ molecules\ cm^{-3}) = 8.6 \times 10^{-11}\ s^{-1} = 3.1 \times 10^{-7}\ h^{-1}$

and

$k'_{O_3} = k_{O_3} \times [O_3] = 2.8 \times 10^{-17}\ cm^3\ molecules^{-1}\ s^{-1} \times 50 \times (2.46 \times 10^{10}\ molecules\ cm^{-3}) = 3.4 \times 10^{-5}\ s^{-1} = 0.12\ h^{-1}$

where 2.46 × $10^{10}$ molecules $cm^{-3}$ is the equivalent molecular concentration of a mixing ratio of 1 ppb at 1 atm and 298 K.

As a result, with the provided first-order mass-transport rates of species out of the KDL, $k_{KDL,out}$ (2.32 $h^{-1}$) in Table S1, the reaction with $O_3$ would only contribute to 1.3×$10^{-5}$ %– 5.2 % of the total decay of the most reactive species in the KDL, and less in other regions of the house. Thus, the levels of $O_3$ injected to the house did not contribute more than 5.2 % to the decay rate of common aromatic compounds when the $O_3$ mixing ratio is at the maximum level of 50 ppb. The same is likely true for the identified insecticide species.

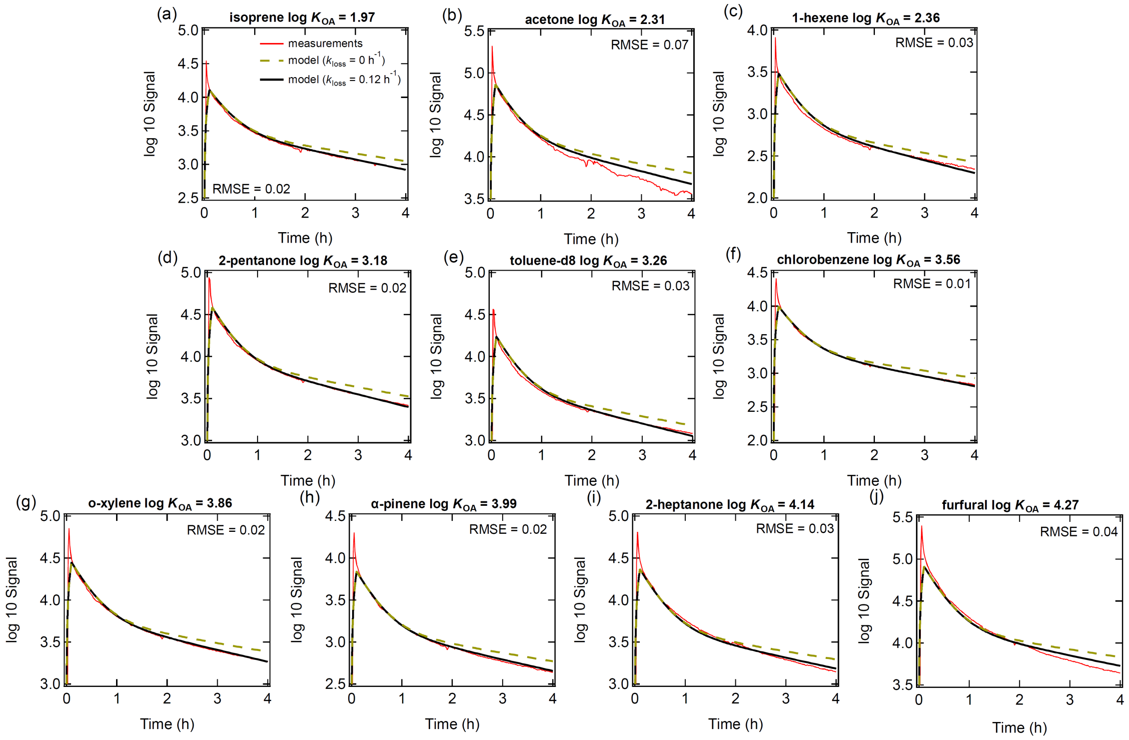


**Fig. S1.** Modelled (black line and dashed brown line) and measured in the KDL (red line) decay profiles for all the CASA cocktail compounds, where the model assumes surface reservoirs are 200 nm in the KDL and 8 μm in the rest of the house. The black model line indicates the impact of including $k_{loss}$ = 0.12 h$^{-1}$ in the model on the fitting. Root mean square error (RMSE) values are shown for the model simulations with $k_{loss}$ = 0.12 h$^{-1}$ and calculated between 30 minutes and 3 hours to exclude data where the house is not well-mixed the signal is low with greater uncertainty. Note that the long-term decays of acetone and furfural are slightly faster than modeled. This may arise from additional interactions with the surface reservoirs, given their more polar nature than the other CASA cocktail compounds.

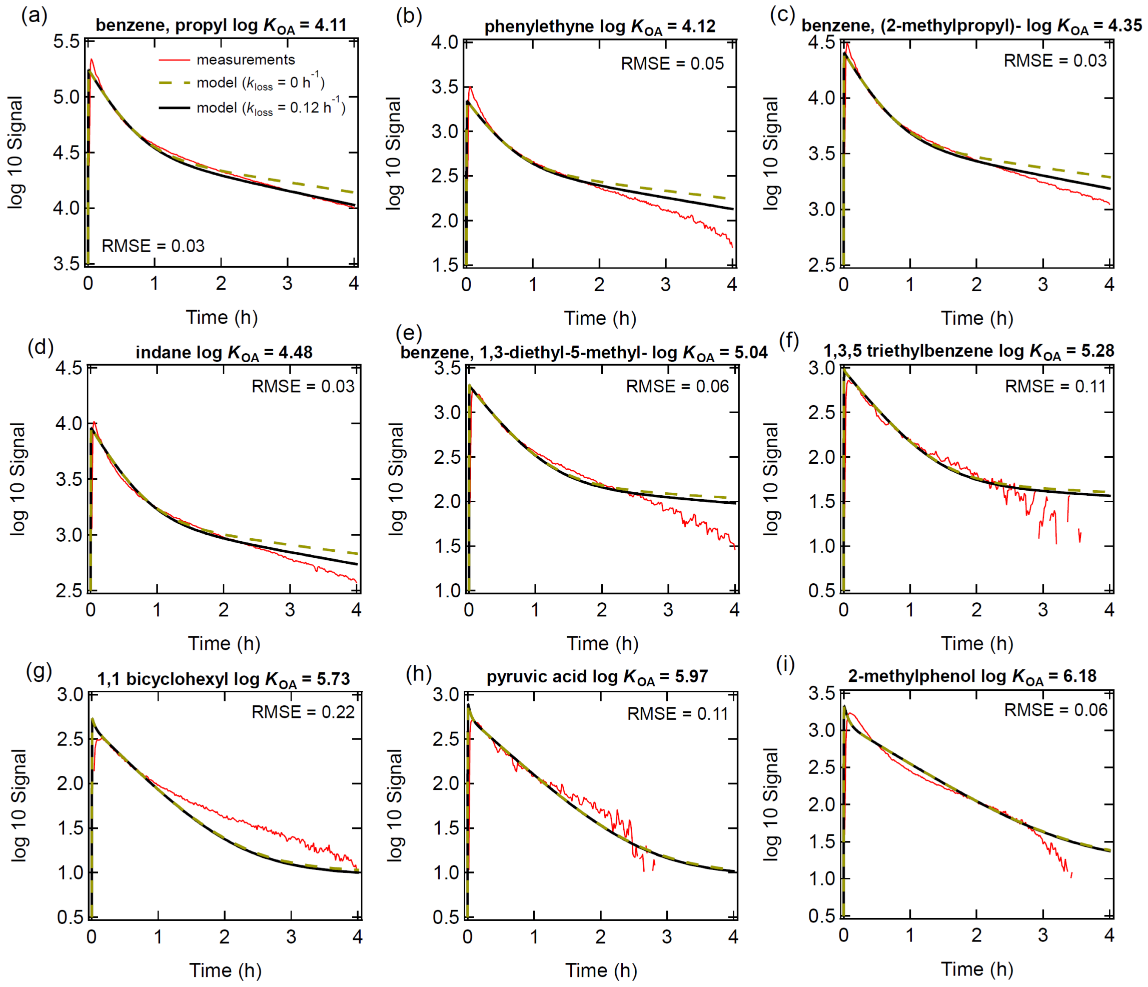


**Fig. S2.** Modelled (black line and dashed brown line) and measured in the KDL (red line) decay profiles for all the insecticide compounds, where the model assumes surface reservoirs are 200 nm in the KDL and 8 μm in the rest of the house. The black model line indicates the impact of including $k_{loss}$ = 0.12 h$^{-1}$ in the model on the fitting. Root mean square error (RMSE) values are shown for the model simulations with $k_{loss}$ = 0.12 h$^{-1}$ and calculated between 30 minutes and 3 hours to exclude data where the house is not well-mixed the signal is low with greater uncertainty.

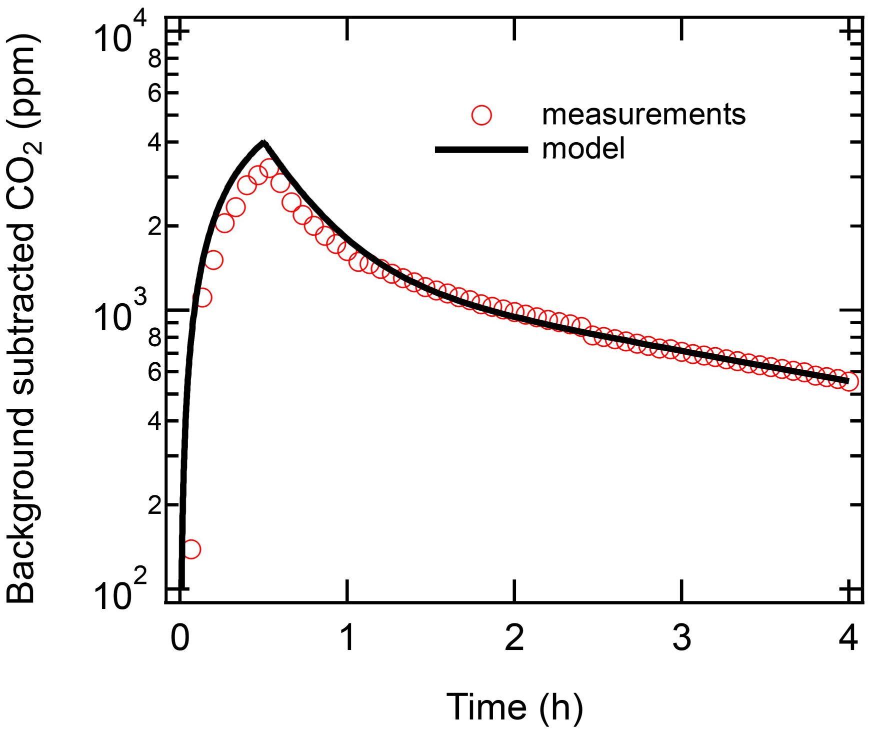


**Fig. S3.** Measurements (red points) of the background subtracted $CO_2$ mixing ratio which was injected into the KDL and modeling results (black line). The fitting region for the model was from ~1 hour (as the $CO_2$ injection lasted for 30 minutes and it takes an additional ~ 30 minutes for the KDL to be well mixed). Details of $CO_2$ injection and measurements were described in Text S4.

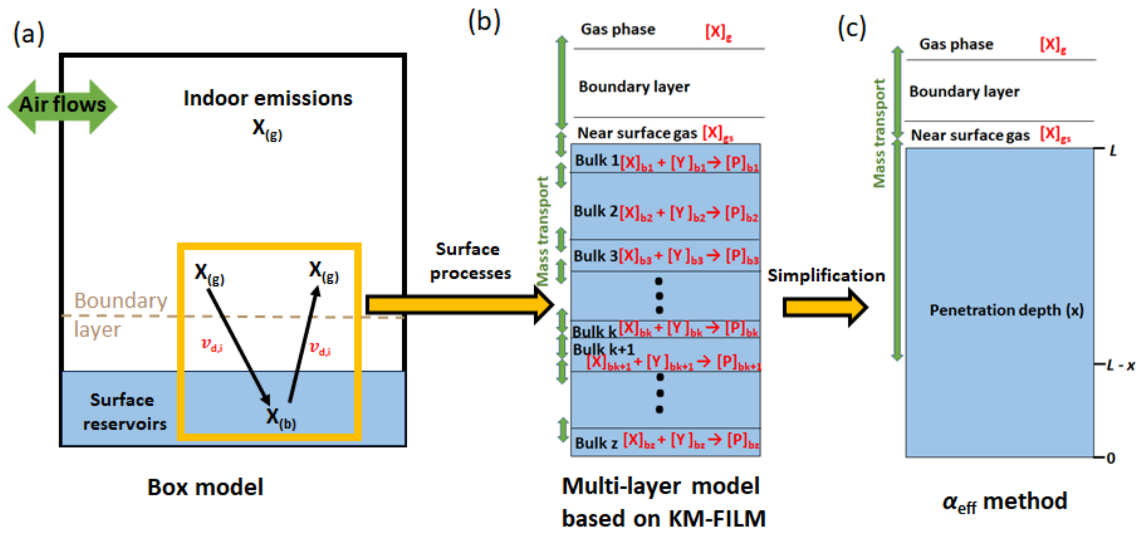


**Fig. S4:** A schematic of the models used in this work. (a) an indoor box model representing processes that can occur in the indoor space including emissions, transport in and out of the indoor space and reversible deposition to surface reservoirs. (b) a kinetic multi-layer model which explicitly treats mass transport from the gas phase to the reservoir surface and Fickian diffusion in the bulk with multiple layers. (c) the simplified $\alpha_{eff}$ method which uses an effective penetration depth to account for mass transport within the surface reservoir.

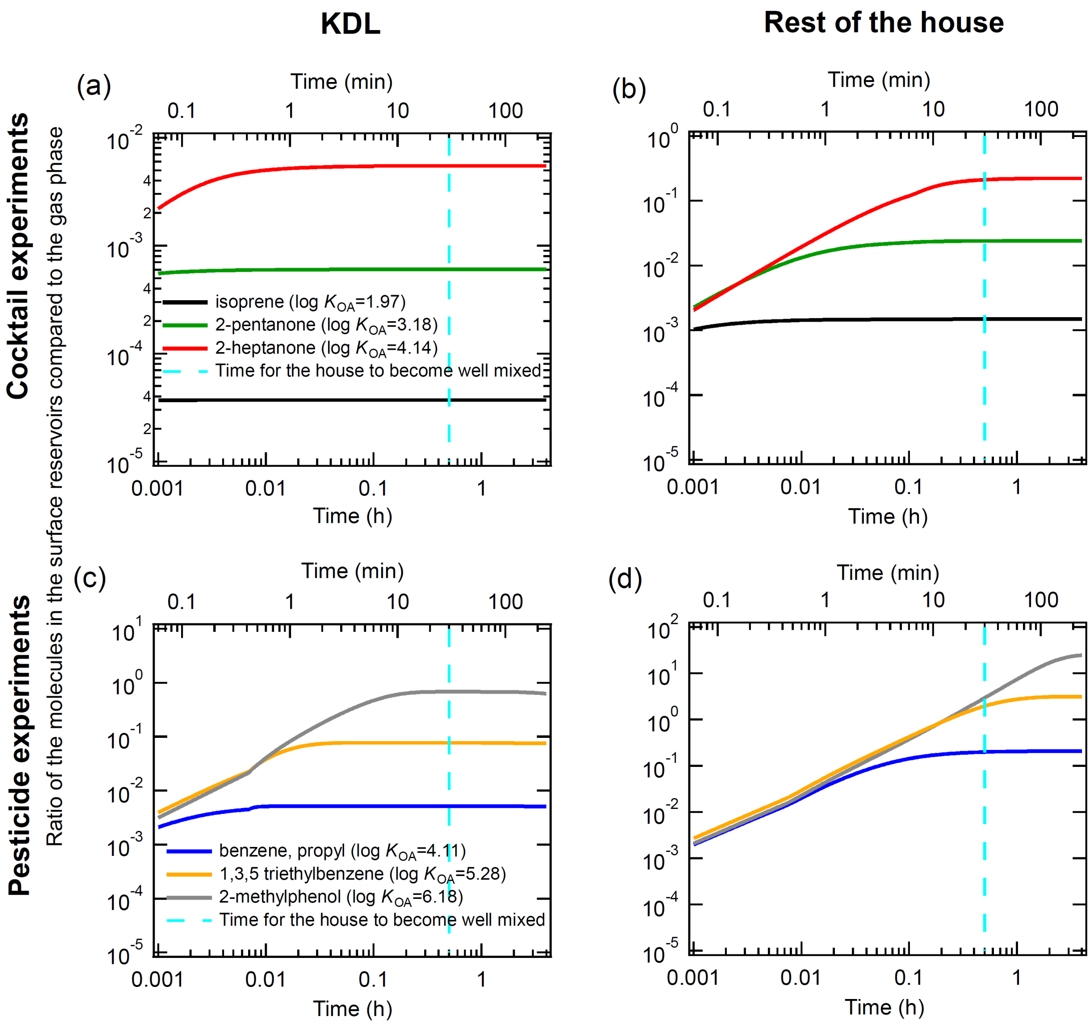


**Fig. S5.** The time evolution of the ratio of molecules in the surface reservoirs compared to the gas phase provided by the model for (a-b) the CASA cocktail experiment and (c-d) the insecticide experiment. Panels (a and c) are for the KDL and panels (b and d) are for the rest of the house. The dashed light blue lines show the time required for the house to become well mixed based on $CO_2$ measurements.

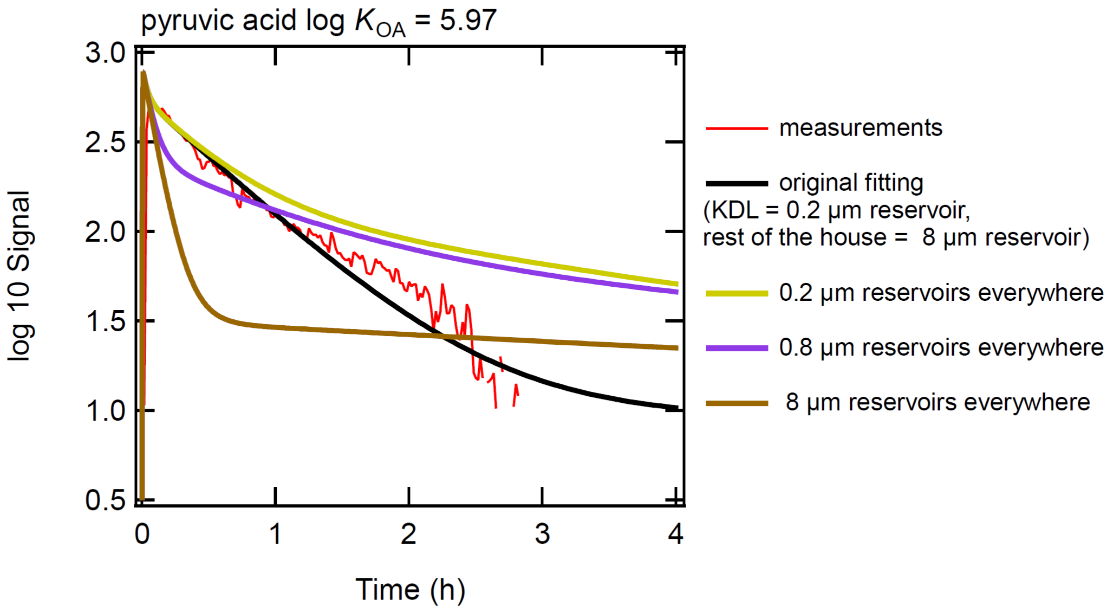


**Fig. S6.** Sensitivity test demonstrating the impact of using the same average octanol-equivalent surface reservoir thicknesses in the model on the decay of pyruvic acid, for the KDL.

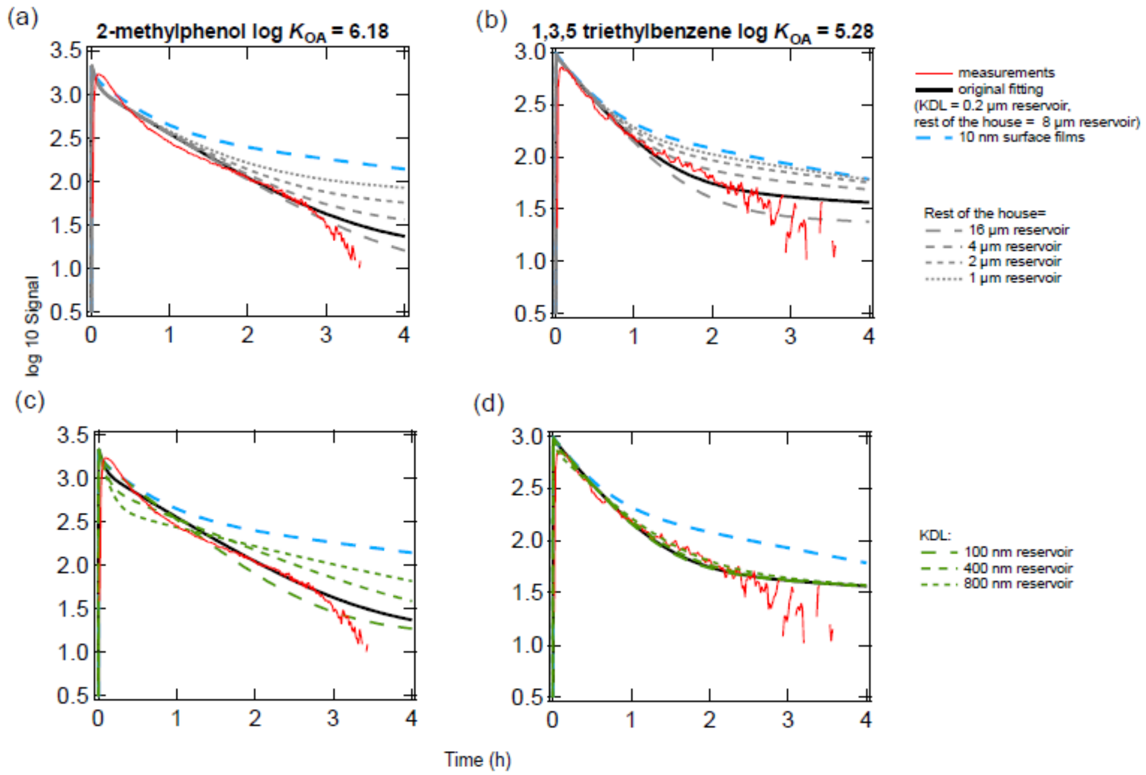


**Fig. S7:** Sensitivity test showing the impact of changing the surface reservoir thickness in (a-b) the rest of the house and (c-d) the KDL for (a, c) 2-methylphenol and (b, d) 1,3,5 trimethylbenzene. These chemicals are shown for illustrative purposes. The depths highlighted in the text (200 nm in the KDL and 8 μm elsewhere) provided the best fit over all eight chemicals.

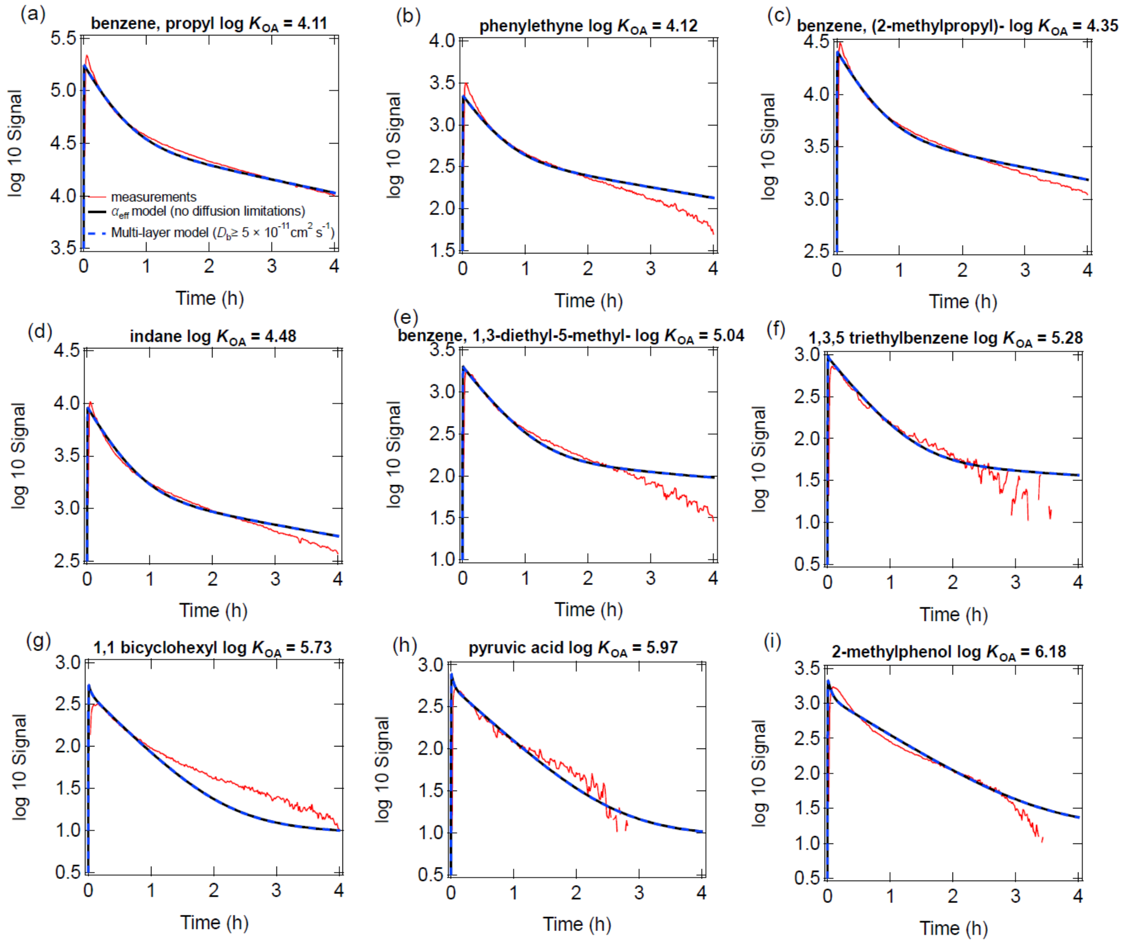


**Fig. S8:** A comparison of the kinetic multi-layer method with $D_b \geq 5 \times 10^{-11}$ cm$^2$ s$^{-1}$ (blue dashed lines) to the $\alpha_{eff}$ effective method (black lines) with $\alpha_{eff} = 1$ (no diffusion limitations) for the insecticide experiments.

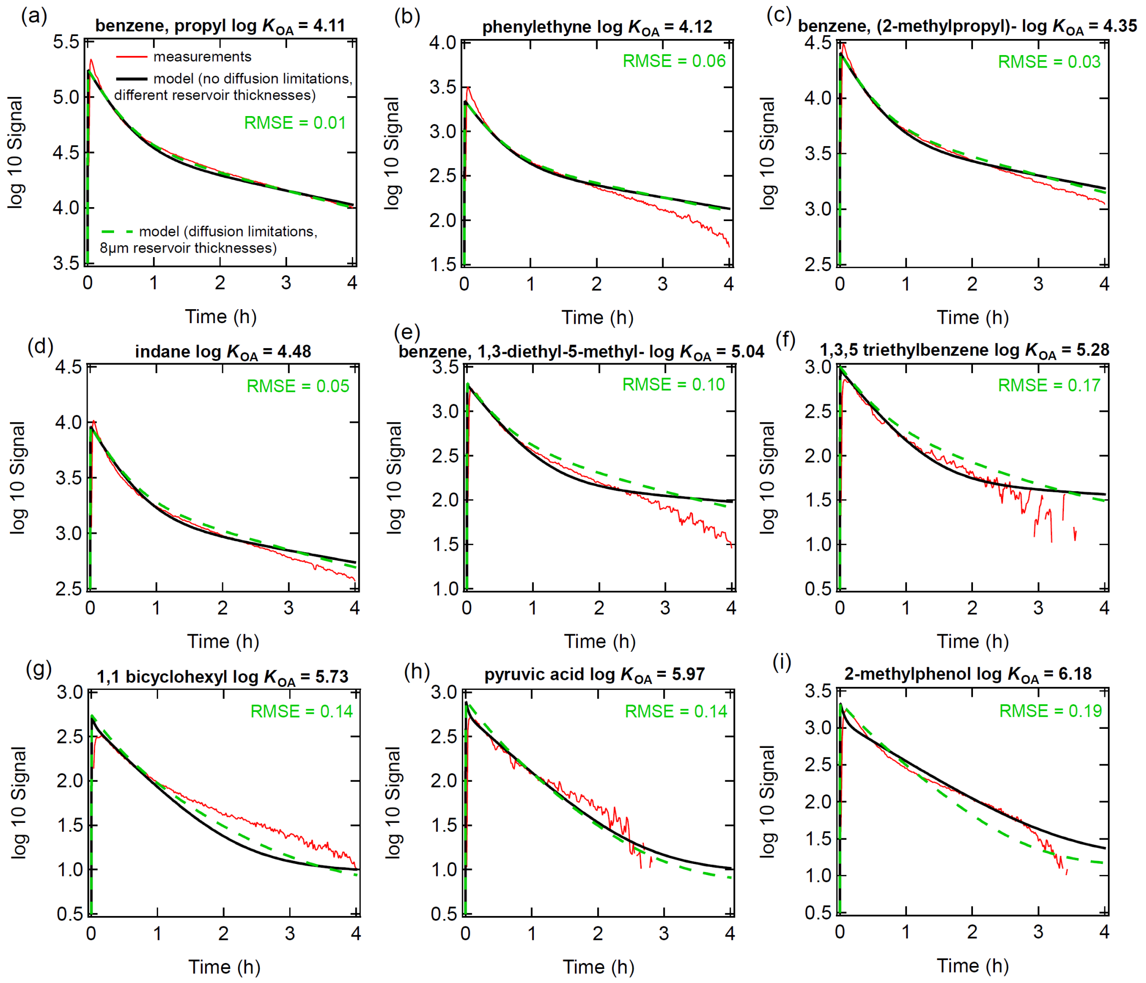


**Fig. S9.** The model fitting to the insecticide measurements with average octanol-equivalent surface reservoir thicknesses of 8μm throughout the whole house, and with bulk diffusion coefficients of $10^{-13}$ cm$^2$ s$^{-1}$ in the KDL and $10^{-11}$ cm$^2$ s$^{-1}$ in the rest of the house. Root mean square error (RMSE) values are shown for the model simulations with diffusion limitations and calculated between 30 minutes and 3 hours to exclude data where the house is not well-mixed and the signal is low with greater uncertainty.

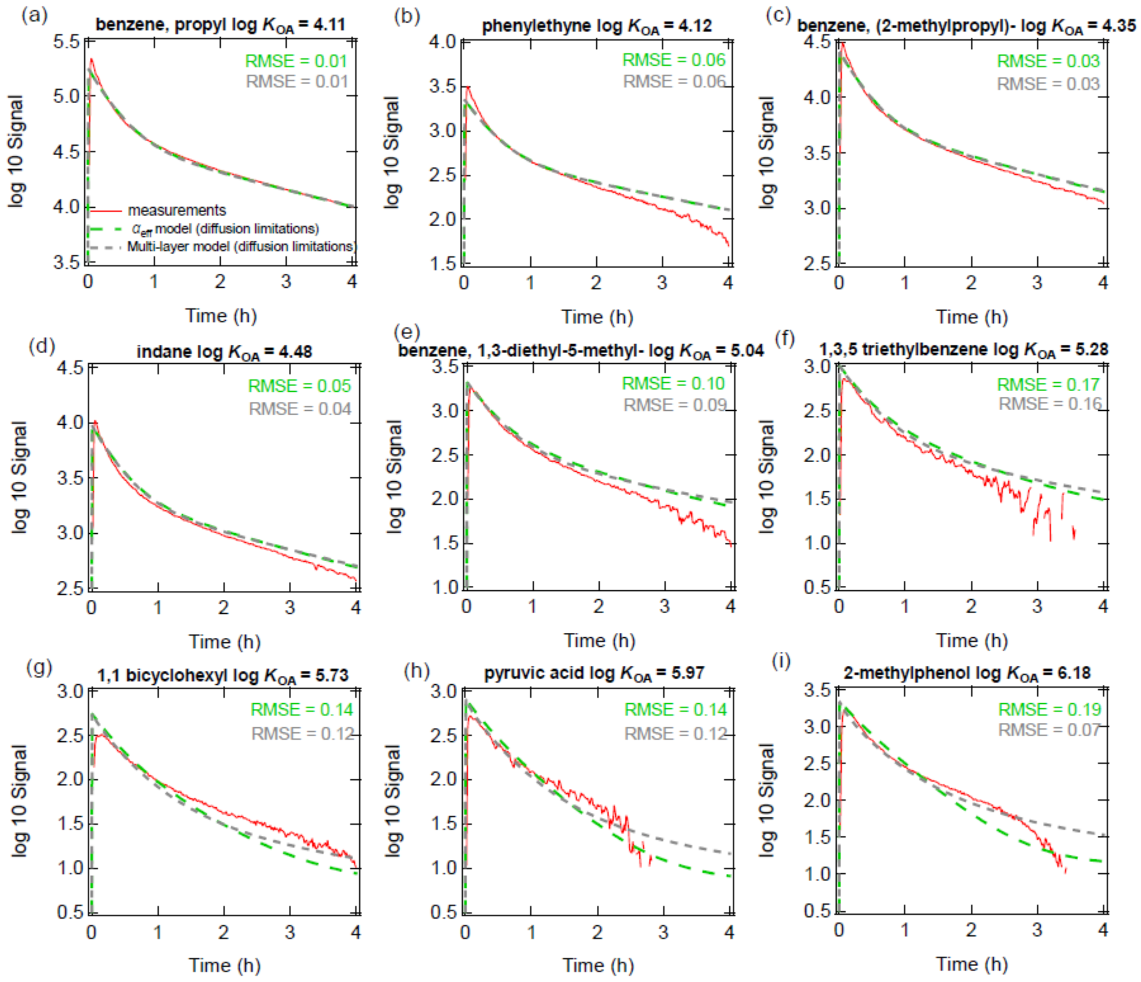


**Fig. S10:** A comparison of the kinetic multi-layer model (grey dashed lines) to the $\alpha_{eff}$ method (green dashed lines) with a surface reservoir thickness of 8μm throughout the house and with $D_b$= $10^{-13}$ $cm^2$ $s^{-1}$ in the KDL and $D_b$ = $10^{-11}$ $cm^2$ $s^{-1}$ in the rest of the house for the insecticide experiments. The slight difference between the fitting for the $\alpha_{eff}$ method and the kinetic multi-layer model for high $K_{OA}$ compounds (f-i) after ~2 h is due to quasi-equilibrium not being reached in the penetration depth for the $\alpha_{eff}$ simulations on the timescale of the experiments.

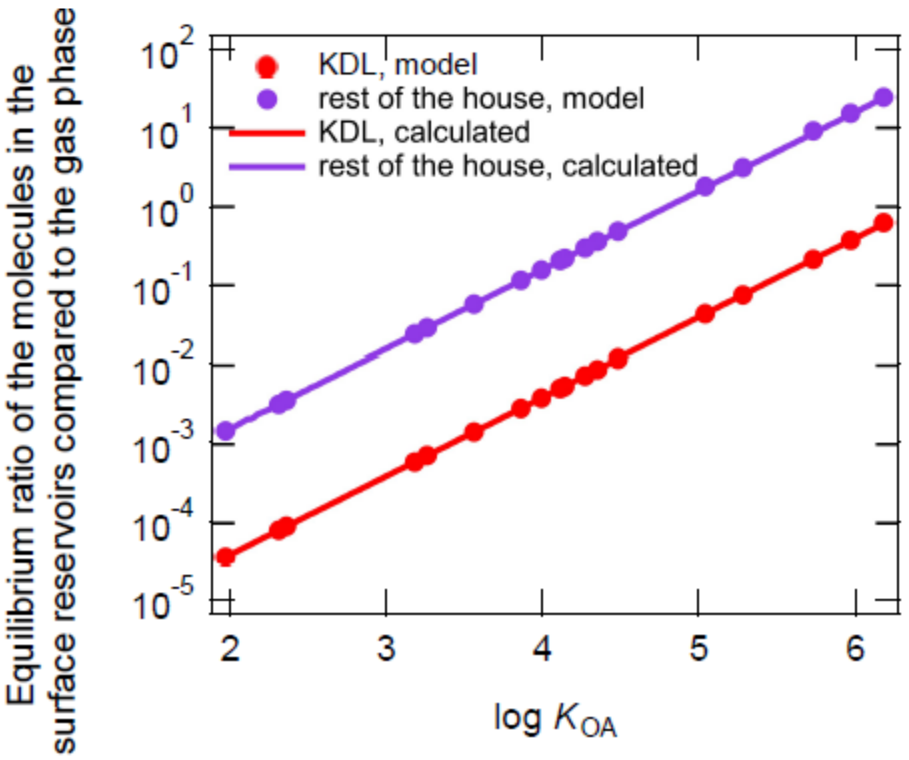


**Fig. S11.** Equilibrium ratios of molecules in the surface reservoirs compared to the gas phase as a function of log $K_{OA}$ values output from the model for each compound (markers) and calculated using a simple equation (lines). Calculations were performed using the equation: Ratio = $\frac{SLK_{OA,X}}{V}$ and model simulations were performed for all compounds used in the cocktail and insecticide experiments. Note that the average octanol-equivalent thickness of surface films ($L$) is 200 nm for the KDL and 8 μm for the rest of the house.

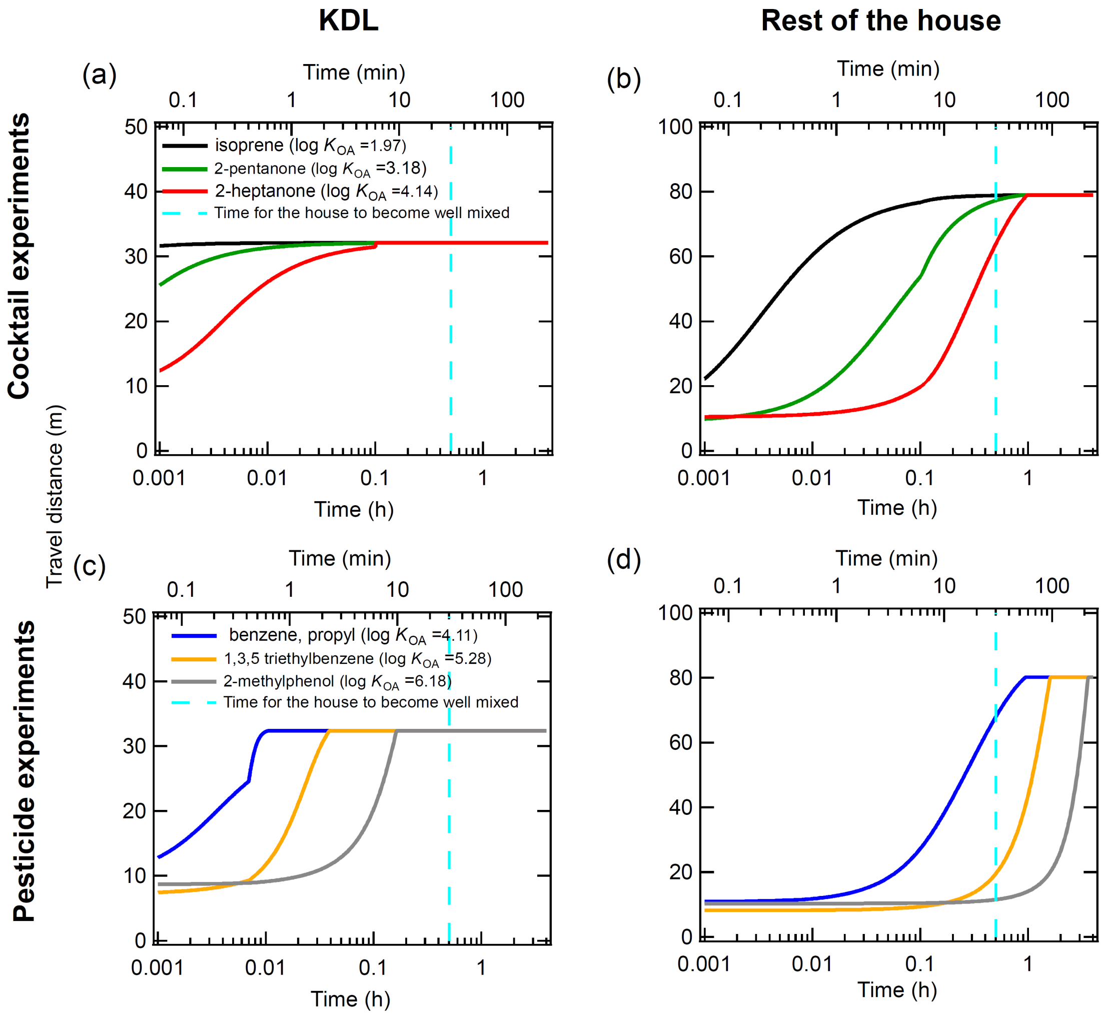


**Fig. S12.** The time evolution of the transport distance of different compounds outputted by the model using equations E9 and E10 for (a-b) the CASA cocktail experiment and (c-d) the insecticide experiment. Panels (a and c) are for the KDL and panels (b and d) are for the rest of the house. The dashed light blue lines show the time required for the house to become well mixed based on $CO_2$ measurements.

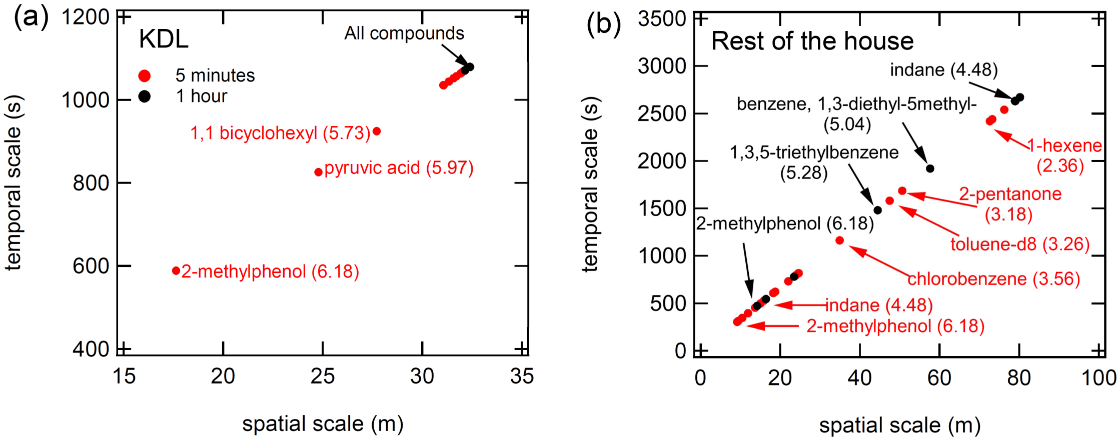


**Fig. S13.** Simulated spatial ($d_t$) and temporal ($t_{\frac{1}{2}}$) scales for compounds used in the cocktail and insecticide experiments in **(a)** the KDL and **(b)** the rest of the house. Red and black markers show the scales 5 minutes and 1 hour after the start of chemical addition, respectively. Log $K_{OA}$ values of compounds are given in the parentheses.

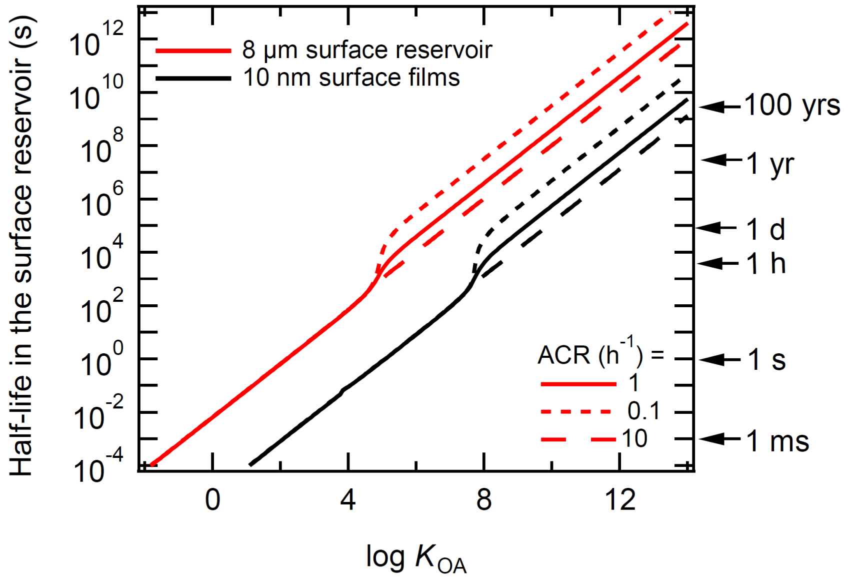


**Fig. S14.** Half-life ($t_{\frac{1}{2}}$) of various compounds in the surface reservoir (with average octanol-equivalent thicknesses of 8 μm (red lines) and 10 nm (black lines)) in the rest of the house as a function of the log $K_{OA}$ value of the compound in the absence of reactions. $u_d$ is set to 3 m h$^{-1}$ for all simulations and three different air change rates (ACR) of 0.1 (short dashed black and red lines), 1 (solid black and red lines), and 10 h$^{-1}$ (long dashed black and red lines) were used in the kinetic model.

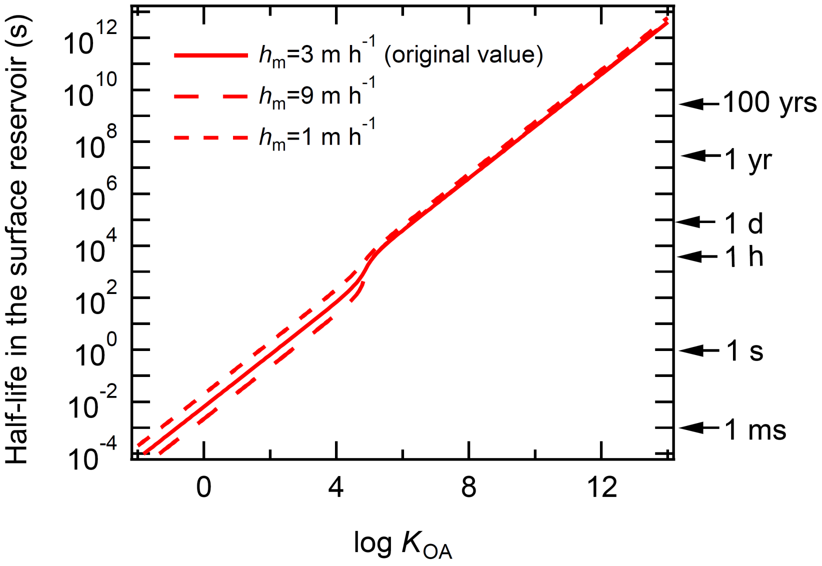


**Fig. S15:** Sensitivity simulations showing the impact of changing the convective mass transfer coefficient by a factor of three on the half-life of a VOC in the surface reservoir.

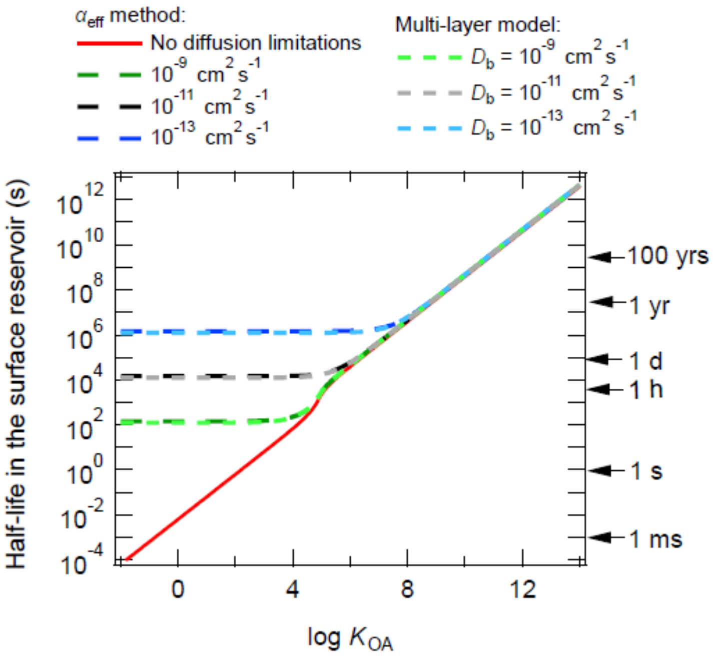


**Fig. S16:** Sensitivity simulations showing the impact of different bulk diffusion coefficients on the half-life of a VOC in a surface reservoir as calculated using the $\alpha_{eff}$ method and the kinetic multi-layer model.

**Table S1.** Volumetric flow rates ($Q$) and volumes ($V$) used to determine first-order transport rates ($k$) of compound $X$ in the kinetic model.

| Parameter | 21st April ($CO_2$ measurements) | 10th March (Cocktail measurements) | 31st March (Insecticide measurements) | Notes and comments |
|---|---|---|---|---|
| $Q_{HRV, Total}$ ($m^3$ $h^{-1}$) | 238 | 238.5 | 232 | Experimentally measured using pitot tube |
| $Q_{ACR,Total}$ ($m^3$ $h^{-1}$) | 241 | 251 | 241 | Experimentally measured at seven locations using daily sulfur hexafluoride tracer decay |
| $Q_{HRV,H,out}$ ($m^3$ $h^{-1}$) | 238 | 238.5 | 232 | Experimentally measured using pitot tube, all HRV exhaust flows come from the non-KDL volume and therefore $Q_{HRV,H,out} = Q_{HRV, Total}$ |
| $Q_{HRV,H,in}$ ($m^3$ $h^{-1}$) | 126 | 126 | 123 | Determined from bolometer measurements of HRV supply register flows at beginning and end of CASA: $Q_{HRV,H,in} = 0.53 \times Q_{HRV, Total}$ $Q_{HRV,KDL,in} = 0.47 \times Q_{HRV, Total}$ |
| $Q_{HRV,KDL,in}$ ($m^3$ $h^{-1}$) | 112 | 112 | 109 | |

| | | | | |
|---|---|---|---|---|
| $Q_{exf,H,out}$ ($m^3$ $h^{-1}$) | 2.34 | 9.75 | 7.02 | Assumed balanced infiltration and exfiltration in each zone and total infiltration/ exfiltration is in ratio to the zone volume.* |
| $Q_{inf,H,in}$ ($m^3$ $h^{-1}$) | 2.34 | 9.75 | 7.02 | |
| $Q_{exf,KDL,out}$ ($m^3$ $h^{-1}$) | 0.66 | 2.75 | 1.98 | |
| $Q_{inf,KDL,in}$ ($m^3$ $h^{-1}$) | 0.66 | 2.75 | 1.98 | |
| $Q_{H,KDL}$ ($m^3$ $h^{-1}$)** | 400 | 400 | 400 | Estimated by fitting to $CO_2$ decay measurements |
| $Q_{KDL,H}$ ($m^3$ $h^{-1}$) | 512 | 512 | 509 | Assumed for flow balance: $Q_{KDL,H}$=0.47 × $Q_{HRV, Total}$ + $Q_{H,KDL}$ |
| $V_H$ ($m^3$)*** | 784 | 784 | 784 | Experimentally measured via tape measure |
| $V_{KDL}$ ($m^3$) | 221 | 221 | 221 | Experimentally measured via tape measure |
| $k_{H,in}$ ($h^{-1}$)*** | 0.65 | 0.65 | 0.65 | Calculated as: $Q_{KDL,H}$/ $V_H$ |
| $k_{H,out}$ ($h^{-1}$) | 0.82 | 0.83 | 0.82 | Calculated as: ($Q_{H,KDL}$ + $Q_{exf,H,out}$ + $Q_{HRV,H,out}$)/ $V_H$ |
| $k_{KDL,in}$ ($h^{-1}$)**** | 1.81 | 1.81 | 1.81 | Calculated as: $Q_{H,KDL}$/ $V_{KDL}$ |
| $k_{KDL,out}$ ($h^{-1}$) | 2.32 | 2.33 | 2.31 | Calculated as: ($Q_{KDL,H}$ + $Q_{exf,KDL,out}$)/ $V_{KDL}$ |

* An infiltration/exfiltration balance is not representative of actual flows. But due to small flow values and low outdoor concentrations this assumption does not impact the final concentrations.

** An assumption is made that this flow does not vary between different days.

*** This volume does not include the attic which mixes with the rest of the house slowly via passive ventilation.

**** Volumetric flow rates into the volumes from outdoors are not included when calculating *k* as the mixing ratio of species *X* outdoors is assumed to be zero.

**Table S2.** Additional parameters used in the kinetic model.

| Parameter | Description | Values | Comments |
|---|---|---|---|
| $L_{KDL}$ | Effective octanol-equivalent average thickness of the surface reservoir in the KDL | 10 nm or 200 nm | Larger thickness values are determined by fitting to the measurement data. Best fits were evaluated with RMSE. Note that the total RMSE of the 5 compounds with log Koa > 5 (which partition significantly into the reservoirs) increases if the reservoir thicknesses are both increased or decreased by more than 10%. Simulations are also performed with 10 nm surface films for comparison. |
| $L_H$ | Effective octanol-equivalent average thickness of the surface reservoir in the rest of the house | 10 nm or 8 $\mu$m | |
| $\alpha_{eff}$ | Effective mass accommodation | 1 | Unless otherwise specifically stated we assume no bulk diffusion limitations which should be the case unless surfaces are quite viscous. |
| $h_m$ | Convective mass transfer coefficient | 3 m $h^{-1}$ | Consistent with the recommended value by Weschler and Nazaroff (19). Note that increasing this value by up to a factor of 10 had no impact on the model fittings to Figure 2 and S9 indicating that VOC uptake and emissions to surfaces were not limited by transport from the air to the surface. |
| $E_{KDL,X}$ | Emission rates | Various in units of $m^{-3}$ $s^{-1}$ | Varied to fit the data. |
| $k_{loss}$ | Unknown first-order loss process which affects all VOCs equally and is determined by fitting to measurements. | 0.12 $h^{-1}$ (VOCs) 0 $h^{-1}$ ($CO_2$) | This is possibly a transport-limited sorption loss. It is a fitting value which is required to explain the faster apparent loss rate of VOCs compared to $CO_2$ at times when the house is well mixed. If the loss occurs in the KDL or in the entire house (rather than just the rest of the house) good fits to the data can be obtained with values of 0.17 $h^{-1}$ and 0.07 $h^{-1}$, respectively. |
| $K_{oa,X}$ | Partitioning coefficient of compounds X | log $K_{OA}$ = 1.97 (isoprene) to log $K_{OA}$ = 6.18 (2-methylphenol) | |

| | | | |
|---|---|---|---|
| $\omega_X$ | Mean thermal velocity of compounds X | Various in units of m $s^{-1}$ | |
| $A_{KDL}/V_{KDL}$ | Surface to volume ratio of the KDL | 2.0 $m^{-1}$ | Assumed to be 2.0 $m^{-1}$.<br>Note that due to uncertainties in these values sensitivity tests have been performed (see text). |
| $A_H/V_H$ | Surface to volume ratio of the rest of the house | 2.0 $m^{-1}$ | |
| $t_{em}$ | Emission time | 6 minutes (CASA cocktail), 25 seconds (insecticide)<br>30 minutes ($CO_2$) | Experimental value |

**Table S3.** The calculated and simulated equilibrium ratio ($\frac{SLK_{OA,X}}{V}$) of molecules in the surface reservoirs compared to the gas phase, as shown for selected compounds in Figure S4. Note that the thickness of surface films ($L$) is 200 nm for the KDL and 8 μm for the rest of the house.

| **Compound Name** | **Log $K_{OA}$*** | **Location** | **Calculated Equilibrium Ratio ($\frac{SLK_{OA,X}}{V}$)** | **Simulated Equilibrium Model Ratio** |
|---|---|---|---|---|
| Isoprene | 1.97 | KDL | $3.7 \times 10^{-5}$ | $3.7 \times 10^{-5}$ |
| 2-pentanone | 3.18 | KDL | $6.0 \times 10^{-4}$ | $6.0 \times 10^{-4}$ |
| Benzene, propyl | 4.11 | KDL | 0.0051 | 0.0051 |
| 2-heptanone | 4.14 | KDL | 0.0055 | 0.0055 |
| 1,3,5-triethylbenzene | 5.28 | KDL | 0.076 | 0.076 |
| 2-methylphenol | 6.18 | KDL | 0.60 | 0.60 |
| | | | | |
| Isoprene | 1.97 | Rest of the house | 0.0015 | 0.0015 |
| 2-pentanone | 3.18 | Rest of the house | 0.024 | 0.024 |
| Benzene, propyl | 4.11 | Rest of the house | 0.21 | 0.21 |
| 2-heptanone | 4.14 | Rest of the house | 0.22 | 0.22 |
| 1,3,5-triethylbenzene | 5.28 | Rest of the house | 3.03 | 3.15** |
| 2-methylphenol | 6.18 | Rest of the house | 24.1 | 25.6** |

* Log $K_{OA}$ values were obtained from ppLFER prediction in the UFZ-LSER database (6).

** Discrepancies between the calculated and modeled ratios can occur due to gas-diffusion limitations across the boundary layer disrupting the equilibrium while other flows remove molecules from the gas-phase (e.g. air-exchange).

**Table S4.** Log $K_{OA}$ value of selected isomers of compounds in the primary compound list.

| Formula | Identity | CAS Number | Log $K_{OA}$ by ppLFER | Model Input Assignment | Note |
|---|---|---|---|---|---|
| $C_3H_4O_3$ | Pyruvic acid | 127-17-3 | 5.97 | √ | |
| | 1,3-Dioxolan-2-one | 96-49-1 | 3.43 | | |
| $C_8H_6$ | Phenylethyne | 536-74-3 | 4.12 | √ | No ppLFER prediction for isomers |
| $C_7H_8O$ | 2-methylphenol | 95-48-7 | 6.18 | √ | |
| | Benzyl alcohol | 100-51-6 | 5.94 | | |
| | p-Cresol | 106-44-5 | 6.46 | | |
| $C_9H_{10}$ | Indane | 496-11-7 | 4.48 | √ | |
| | Benzene, 1-ethenyl-3-methyl- | 100-80-1 | 4.29 | | |
| | trans-β-Methylstyrene | 873-66-5 | 4.38 | | |
| $C_{12}H_{18}$ | 1,3,5-triethylbenzene | 102-25-0 | 5.28 | √ | |
| | Benzene, hexyl- | 1077-16-3 | 5.51 | | |
| | Benzene, 1,4-bis(1-methylethyl)- | 100-18-5 | 5.15 | | |

**Table S5.** Final combined list of identified compounds for insecticide spray can.

| Formula | Assigned Identity | CAS Number | Log $K_{OA}$ by ppLFER | Source (primary / secondary list) | Observed in GC-MS? If yes, matching %? |
|---|---|---|---|---|---|
| $C_3H_4O_3$ | pyruvic acid | 127-17-3 | 5.97 | Primary | No |
| $C_8H_6$ | Phenylethyne | 536-74-3 | 4.12 | Primary | No |
| $C_7H_8O$ | 2-methylphenol | 95-48-7 | 6.18 | Primary | No |
| $C_9H_{10}$ | Indane | 496-11-7 | 4.48 | Primary | Yes* |
| $C_9H_{12}$ | Benzene, propyl | 103-65-1 | 4.11 | Primary | Yes (85%) |
| $C_{10}H_{14}$ | Benzene, (2-methylpropyl)- | 538-93-2 | 4.35 | Primary | Yes (80%) |
| $C_{11}H_{16}$ | Benzene, 1,3-diethyl-5-methyl- | 2050-24-0 | 5.04 | Secondary | Yes (90%) |
| $C_{12}H_{18}$ | 1,3,5-triethylbenzene | 102-25-0 | 5.28 | Primary | No |
| $C_{12}H_{22}$ | 1,1'-Bicyclohexyl | 92-51-3 | 5.73 | Secondary | Yes (65%) |

* Formula assigned by NIST library is likely inaccurate after a manual inspection of collected MS. Therefore, the identity provided by the PTR-library is used.

**SI References**


1. J. Krechmer, *et al.*, Evaluation of a New Reagent-Ion Source and Focusing Ion–Molecule Reactor for Use in Proton-Transfer-Reaction Mass Spectrometry. *Anal Chem* **90**, 12011–12018 (2018).
2. P. Mochalski, *et al.*, PTR-MS studies of the reactions of H3O+ with a number of deuterated volatile organic compounds and the subsequent sequential reactions of the primary product ions with water under normal and humid drift tube conditions: Implications for use of deuterated compounds for breath analysis. *Int J Mass Spectrom* **436**, 65–70 (2019).
3. D. Pagonis, K. Sekimoto, J. de Gouw, A Library of Proton-Transfer Reactions of H3O+ Ions Used for Trace Gas Detection. *J Am Soc Mass Spectrom* **30**, 1330–1335 (2019).
4. X. Wang, A. W. H. Chan, Particulate Matter and Volatile Organic Compound Emissions Generated from a Domestic Air Fryer. *Environ Sci Technol* **57**, 17384–17392 (2023).
5. P. J. Linstorm, W. G. Mallard, *NIST Chemistry Webbook, NIST Standard Reference Database Number 69* (1998).
6. N. Ulrich, *et al.*, UFZ - LSER Database. (2017). Available at: https://www.ufz.de/index.php?en=31698&contentonly=1&m=0&lserd_data[mvc]=Public/start [Accessed 21 March 2024].
7. P. S. J. Lakey, B. E. Cummings, M. S. Waring, G. C. Morrison, M. Shiraiwa, Effective mass accommodation for partitioning of organic compounds into surface films with different viscosities. *Environ Sci Process Impacts* **25**, 1464–1478 (2023).
8. C. J. Weschler, W. W. Nazaroff, Growth of organic films on indoor surfaces. *Indoor Air* **27**, 1101–1112 (2017).
9. M. Shiraiwa, U. Pöschl, Mass accommodation and gas–particle partitioning in secondary organic aerosols: dependence on diffusivity, volatility, particle-phase reactions, and penetration depth. *Atmos Chem Phys* **21**, 1565–1580 (2021).
10. P. S. J. Lakey, C. M. A. Eichler, C. Wang, J. C. Little, M. Shiraiwa, Kinetic multi-layer model of film formation, growth, and chemistry (KM-FILM): Boundary layer processes, multi-layer adsorption, bulk diffusion, and heterogeneous reactions. *Indoor Air* **31**, 2070–2083 (2021).
11. M. Shiraiwa, C. Pfrang, T. Koop, U. Pöschl, Kinetic multi-layer model of gas-particle interactions in aerosols and clouds (KM-GAP): linking condensation, evaporation and chemical reactions of organics, oxidants and water. *Atmos Chem Phys* **12**, 2777–2794 (2012).
12. M. Shiraiwa, C. Pfrang, U. Pöschl, Kinetic multi-layer model of aerosol surface and bulk chemistry (KM-SUB): the influence of interfacial transport and bulk diffusion on the oxidation of oleic acid by ozone. *Atmos Chem Phys* **10**, 3673–3691 (2010).
13. P. S. J. Lakey, *et al.*, Spatial and temporal scales of variability for indoor air constituents. *Commun Chem* **4**, 110 (2021).
14. G. Morrison, P. S. J. Lakey, J. Abbatt, M. Shiraiwa, Indoor boundary layer chemistry modeling. *Indoor Air* **29**, 956–967 (2019).

15. J. Li, *et al.*, The persistence of smoke VOCs indoors: Partitioning, surface cleaning, and air cleaning in a smoke-contaminated house. *Sci Adv* **9**, eadh8263 (2023).
16. C. T. Pate, R. Atkinson, J. N. Pitts, The gas phase reaction of O3 with a series of aromatic hydrocarbons. *J Environ Sci Health A Environ Sci Eng* **11**, 1–10 (1976).
17. R. Atkinson, S. M. Aschmann, D. R. Fitz, A. M. Winer, J. N. Pitts, Rate constants for the gas-phase reactions of O3 with selected organics at 296 K. *Int J Chem Kinet* **14**, 13–18 (1982).
18. A. Tomas, R. I. Olariu, I. Barnes, K. H. Becker, Kinetics of the reaction of O3 with selected benzenediols. *Int J Chem Kinet* **35**, 223–230 (2003).
19. C. J. Weschler, W. W. Nazaroff, Semivolatile organic compounds in indoor environments. *Atmos Environ* **42**, 9018–9040 (2008).
